# Photoreceptor laminin drives differentiation of human pluripotent stem cells to photoreceptor progenitors that partially restore retina function

**DOI:** 10.1016/j.ymthe.2022.12.012

**Published:** 2023-01-12

**Authors:** Hwee Goon Tay, Helder Andre, Vicki Chrysostomou, Swarnaseetha Adusumalli, Jing Guo, Xiaoyuan Ren, Wei Sheng Tan, Jia En Tor, Aida Moreno-Moral, Flavia Plastino, Hammurabi Bartuma, Zuhua Cai, Sai Bo Bo Tun, Veluchamy Amutha Barathi, Gavin Tan Siew Wei, Gianluca Grenci, Li Yen Chong, Arne Holmgren, Anders Kvanta, Crowston Jonathan Guy, Enrico Petretto, Karl Tryggvason

**Affiliations:** 1 Centre for Vision Research, Duke-NUS Medical School, Singapore; 2 Cardiovascular and Metabolic Disorders Program, Duke-NUS Medical School, Singapore; 3 Department of Clinical Neuroscience, St. Erik Eye Hospital, Karolinska Institutet, Stockholm, Sweden; 4 Academic Clinical Program, Duke-NUS Medical School, Singapore; 5 Department of Medical Biochemistry and Biophysics, Karolinska Institutet, Stockholm, Sweden; 6 Singapore Eye Research Institute, Singapore National Eye Centre, Singapore; 7 Department of Ophthalmology, Yong Loo Lin School of Medicine, National University of Singapore, Singapore; 8 Mechanobiology Institute (MBI) and Department of Biomedical Engineering, NUS, Singapore; 9 Division of Nephrology, Department of Medicine, Duke University, Durham, NC, USA

**Keywords:** retina-specific laminin isoform, pluripotent embryonic stem cells, photoreceptor progenitors, synaptic connectivity, cell-based therapeutics

## Abstract

Blindness caused by advanced stages of inherited retinal diseases and age-related macular degeneration are characterized by photoreceptor loss. Cell therapy involving replacement with functional photoreceptor-like cells generated from human pluripotent stem cells holds great promise. Here, we generated a human recombinant retina-specific laminin isoform, LN523, and demonstrated the role in promoting the differentiation of human embryonic stem cells into photoreceptor progenitors. This chemically defined and xenogen-free method enables reproducible production of photoreceptor progenitors within 32 days. We observed that the transplantation into *rd10* mice were able to protect the host photoreceptor outer nuclear layer (ONL) up to 2 weeks post transplantation as measured by full-field electroretinogram. At 4 weeks post transplantation, the engrafted cells were found to survive, mature, and associate with the host’s rod bipolar cells. Visual behavioral assessment using the water maze swimming test demonstrated visual improvement in the cell-transplanted rodents. At 20 weeks post transplantation, the maturing engrafted cells were able to replace the loss of host ONL by extensive association with host bipolar cells and synapses. Post-transplanted rabbit model also provided congruent evidence for synaptic connectivity with the degenerated host retina. The results may pave the way for the development of stem cell-based therapeutics for retina degeneration.

## Graphical abstract

**Figure:**
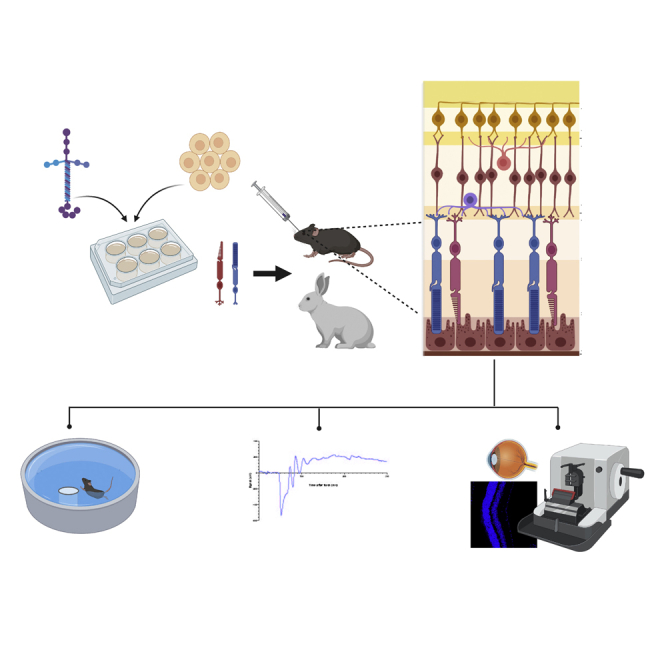

## Teaser

Loss of photoreceptor cells in the eye could lead to blindness. Cell therapy holds promise for treatment by replacement of healthy photoreceptor-like cells. We have developed a method to produce photoreceptor progenitors that could mature in the eyes and that improved visual indications after transplantation in pre-clinical animal models.

## Introduction

Age-related macular degeneration (AMD) and retinitis pigmentosa are examples that are characterized by the progressive and irreversible loss of photoreceptor cells in the retina. These retinal diseases are the major cause of blindness worldwide.^1^^,^^2^ Vision loss severely compromises the quality of life and contributes to extensive socioeconomic burden. The human retina lacks the ability to regenerate cells lost in disease or injury, and, currently, no treatments exist that can restore the lost photoreceptors. Despite promising advancements in gene therapy methods, they can be employed only to induce transgene expression in viable photoreceptor cells, but not to replace lost cells.^3^^,^^4^ Hence, the transplantation of photoreceptors represents a potentially viable therapeutic approach that would replace lost cells with healthy ones expressing proteins crucial for vision. Stem cell-derived retinal organoids could provide healthy photoreceptors for use in cell replacement therapy. As such, several recent studies have described the transplantation of pre-organized differentiated retinal sheets,^5^ optogenetically engineered photoreceptors,^6^ or purified GFP-expressing cone (L/Mopsin) photoreceptors^7^ that were obtained from retinal organoids differentiated for 64, 70, and 119 days respectively. Importantly, the transplanted cells were shown to promote visual function by establishing connectivity with the host rod bipolar cells located in the inner retina. In these studies, microelectroretinogram analysis using multi-electrode array (MEA), instead of full-field electroretinogram (ERG), were used to measure the targeted light-evoked spiking activity of the enucleated retina explant containing localized cell engraftment site. However, retinal organoids are complex tissue structures that exhibit batch-to-batch variations and limited reproducibility, requiring long cell differentiation duration. Their generation also mainly requires the use of chemically undefined non-human components such as animal sera and mouse tumor-derived Matrigel.^8^^,^^9^^,^^10^^,^^11^^,^^12^^,^^13^^,^^14^^,^^15^^,^^16^^,^^17^^,^^18^^,^^19^ All these factors may limit the possibility of using organoids in human cell replacement therapy. Therefore, there remains a need for faster and more efficient methods that can facilitate the directed differentiation of stem cells into photoreceptors.

Laminins are a large family of heterotrimeric basement membrane (BM) proteins present at the basal surfaces of all epithelial and endothelial cells, as well in the ultra-thin BMs that surround single cells, such as photoreceptors, cardiomyocytes, muscle fibers, and adipocytes.^20^ There are five different α chains (LAMA1, LAMA2, LAMA3, LAMA4, LAMA5), four different β chains (LAMB1, LAMB2, LAMB3, LAMB4) and three γ chains (LAMC1, LAMC2, LAMC3). Each isoform contains one α, one β, and one γ chain,^20^^,^^21^ giving rise to a total of 16 different laminin isoforms in mammals, including humans. The different isoforms exhibit varying degrees of tissue-specific locations. Of these, specific isoforms have different effects on the capacities of pluripotent stem cells to be differentiated into different cell types; e.g., to endothelial cells, cardiomyocytes, keratinocyte stem cells, and dopamine neurons.^22^^,^^23^^,^^24^^,^^25^ Furthermore, seven of these laminin isoforms have been found to be enriched in the retina^21^^,^^26^: LN523, LN323, and LN332 are present in the matrix surrounding the rod and the cone photoreceptors,^21^^,^^26^ in the brain, as well as in the BM of testicular seminiferous tubules^27^^,^^28^; LN511, LN521, LN111, LN121, and LN523 are present in different proportions in the Bruch’s membrane located immediately beneath the retinal pigmented epithelial (RPE) layer.^21^^,^^26^

Here, we describe an approach for the directed *in vitro* differentiation of human embryonic stem cells (hESCs) to photoreceptor progenitors under xeno-free, chemically defined conditions. We generated a recombinant protein, human retina-specific laminin isoform LN523, and cultured hESCs using this protein. Since this gamma 3 chain-containing laminin isoform has been reported to be particularly enriched in the retina and brain,^26^^,^^27^^,^^28^^,^^29^^,^^30^ we hypothesized that its presence could recapitulate the retina matrix niche environment, supporting the differentiation of pluripotent hESCs into the photoreceptor lineage. Our results showed that the incorporation of LN523 in the matrix indeed drove the differentiation of hESCs into photoreceptor progenitors, as early as after 32 days in culture. We also showed that the hESC-derived photoreceptor progenitor cells engrafted in the genetic *rd10* retina degeneration rodent model as well as inducing degenerated retina of rabbits. In this study, we used full-field ERG instead of MEA to investigate the functional recovery of the transplanted rodent retina without enucleation. Despite cell engraftment in a confined targeted host retina region, short-term functional recovery was still detected. The transplanted human photoreceptor progenitors expressed post-mitotic mature photoreceptor markers (rhodopsin and opsin) and exhibited synaptic connectivity with the host’s inner retina after 3 to 5 months post transplantation.

## Results

### Recombinant human LN523 supports differentiation to photoreceptor progenitors

**Figure 1:**
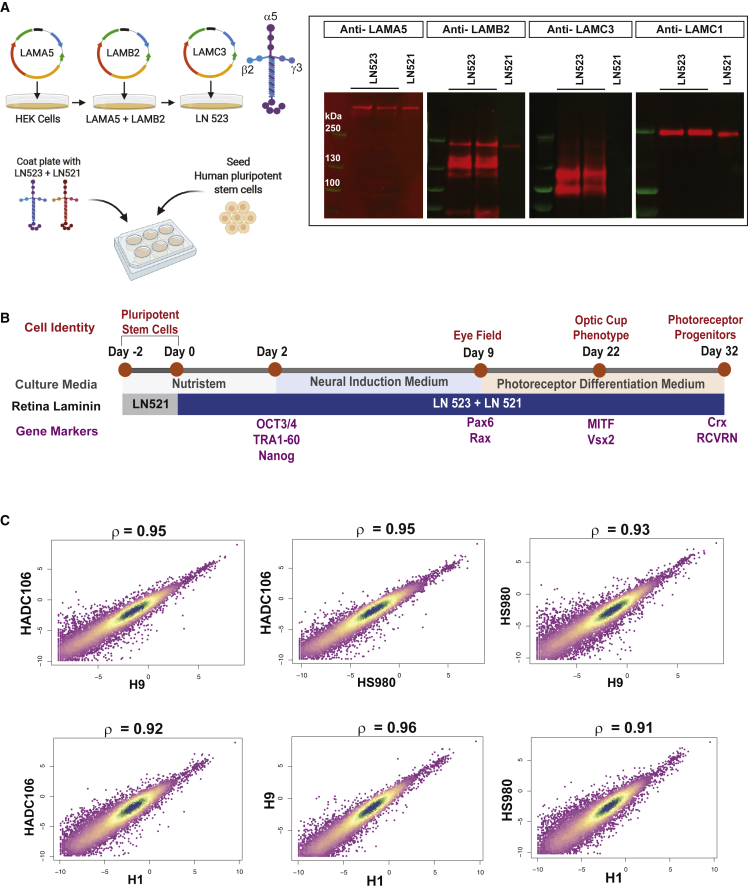
Production of recombinant human retina laminin isoform LN523 and its use in photoreceptor differentiation method showing high reproducibility (A) Schematic description of the synthesis of human recombinant retina-specific laminin isoform LN523 in human embryonic kidney cells (HEK293). HEK293 cells were sequentially transfected with plasmids expressing individual laminin chains. Western blot analyses showing α5 (LAMA5), β2 (LAMB2), and γ3 (LAMC3) chains in purified fractions of LN523 after ion-exchange and gel-filtration chromatography. (B) LN523-based photoreceptor differentiation procedure in human embryonic stem cells (hESCs). Pluripotent hESCs were transferred from LN521-coated plates to plates coated with LN523 + LN521 in NutriStem medium. The medium was changed to neural induction medium on day 2 and to photoreceptor differentiation medium on day 9. (C) Reproducibility of the photoreceptor differentiation protocol is high across four hESC lines (H1, H9, HS980, and HADC106). Correlation of single-cell transcriptome expression levels across the cell lines at day 32. Each point in the scatterplot represents a single gene (expression averaged across cells in each cell line). In each plot, the Spearman’s rank correlation coefficient is indicated as ρ (rho) and the p values for all the pairwise correlation comparisons are <2.2 × 10^−16^.

We hypothesized that LN523 and LN323 may be involved in the differentiation of pluripotent stem cells into photoreceptor cells. The recapitulation of the inter-photoreceptor matrix niche may prime or condition the hESCs toward the retinal neural lineage. To test this, we produced LN523 and LN323 as recombinant human proteins using essentially the same method as described in our previous studies.^31^^,^^32^ Briefly, human embryonic kidney (HEK) 293 cells were first sequentially transfected with expression vectors for LN523 and LN323 component chains (Figure 1A), after which cell clones expressing the highest levels of LN523 and LN323 were isolated and expanded. The cell culture media of the expanded clones were purified by dialysis, gel-filtration, and anionic ion-exchange chromatography. The purified fractions were subsequently analyzed by Coomassie blue staining of SDS-PAGE gels and western blots to identify clones that produce laminin α5, α3, β2, and γ3 chains (Figures 1A, S1A, and S1D; section “materials and methods”). Furthermore, we used mass spectrophotometry to confirm the identities of LN523 and LN323 (Table S1). We also observed that minor amounts of the LN521 isoform (4% compared with LN523) were endogenously produced by HEK293 cells. LN521 is a ubiquitous BM laminin that supports adhesion and expansion of human pluripotent stem cells and all basement membrane-associated cell types *in vivo*.^33^^,^^34^ The full-length laminin γ3 chain (NP_006050) in LN523 contains 1,575 amino acid residues, whereas the full-length γ1 chain (NP_002284) in LN521 contains 1,609 residues. Notably, the γ3 chain lacks 23 amino acids at the N terminus compared with the γ1 chain, and their amino acid sequence identity is only 43.8% based on Waterman-Eggert score obtained from ClustalW pairwise alignment analysis. Therefore, LN523 and LN521 are genetically distinct, although the biological effects of these differences are still unknown.

Next, we studied whether the addition of human recombinant LN523 to the cell culture matrix that contains LN521 influenced the differentiation of hESCs into photoreceptor progenitors. On day 0, the hESCs were plated on a mixture of LN523 + LN521 (2:1 ratio) (Figure 1B). On day 2, the culture medium was changed to neural induction medium for 7 days, after which it was changed to photoreceptor differentiation medium for the remainder of the experiment until day 32 (see section “materials and methods”; Figure 1B). We observed morphological changes in the pluripotent hESCs over the course of 32 days that were indicative of their differentiation into the neural retina lineage that comprises photoreceptor progenitors (Figure S2A). Additionally, karyotype analysis showed that the photoreceptor progenitors did not develop major chromosomal abnormalities during that time period (Figure S2B). To further study the reproducibility of this laminin LN523-based photoreceptor differentiation method, we tested it using four other pluripotent stem cell lines: H1, H9, HS980, and HADC106. These cell lines were also initially maintained in pluripotent form in NutriStem medium. Subsequently, these hESC lines were differentiated similarly on cell culture matrix containing LN523 + LN521. Highly reproducible results were observed between these hESC lines, as shown by the correlation of single-cell whole-transcriptome expression levels at day 32 (Figure 1C); ρ (rho) values for all the pairwise correlation comparisons ranged from 0.91 to 0.96 (p < 2.2 × 10^−16^). Furthermore, the dot plot quantification analysis also showed the expression of photoreceptor progenitor/rod-specific genes in these embryonic cell lines respectively (Figure S2C).

**Figure 2:**
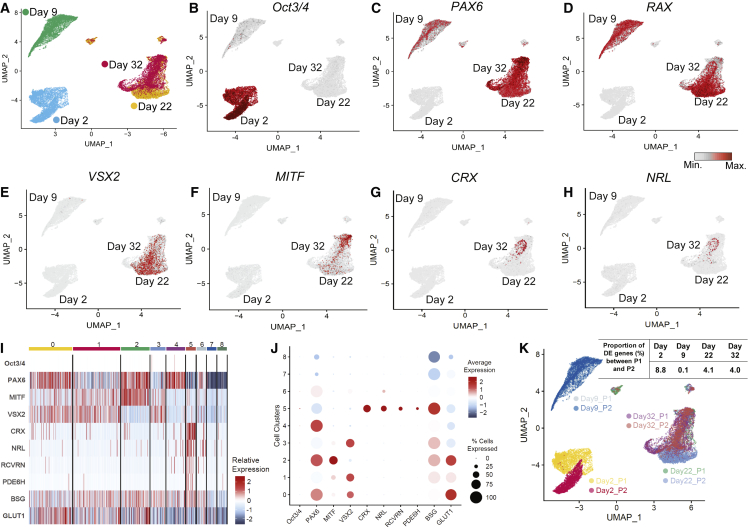
Single-cell transcriptome analyses for the characterization of photoreceptor cells differentiating on LN523 at various time points (A) Visualization of single-cell cluster transcriptomes in the Uniform Manifold Approximation and Projection (UMAP) space (a dimension reduction technique) allowed the separation of cells into distinct clusters, which were mapped to different time points of LN523 + LN521 cultured H1 hESCs. (B) *Oct3/4* pluripotency markers are strongly expressed on day 2 (red) but not on day 9, day 22, or day 32 (light gray). The expression of eye-field markers *Pax6* (C) and *RAX* (D) intensifying from day 9 to day 32. Optic cup markers *Vsx2* (E) and *MITF* (F) are highly upregulated on day 22. The photoreceptor progenitor marker *CRX* (G) and the rod precursor *Nrl* (H) are enriched on day 32. (I) Heatmap of single-cell expression for selected cell identity marker genes in the nine cell clusters (0–8) identified at days 22 and 32. Cells expressing markers of photoreceptor progenitors are found predominantly in cluster 5 at day 32 (also see Figure S3). (J) For each of the nine cell clusters (0–8) identified at days 22 and 32, dot plot quantification analysis shows the percentage of cells expressing different cell identity marker genes, including rod and cone precursors and BSG receptor. The color of each marker gene is indicative of its average gene expression calculated across all cells within a specific cluster. (K) Reproducibility of the photoreceptor differentiation protocol is assessed by similarities in single-cell transcriptomic profiles in the two passages (P1 and P2) of H1 hESC, which is represented in the UMAP space. The proportions of differentially expressed (DE) genes (adjusted p value <5%) between P1 and P2 passages at each time point are shown in the table. The proportion of DE genes is calculated with respect to the total number of genes expressed in both P1 and P2 at each time point (see section “materials and methods”).

Single-cell RNA-sequencing (RNA-seq) analyses on differentiated H1 hESCs revealed distinct cell clusters expressing specific developmental markers at different time points (Figure 2A). The pluripotency markers *Oct3/4* and *Nanog* were strongly expressed in cells cultured on LN523 matrix at day 0 but were gradually downregulated, with low levels of expression at day 9 (Figure 2B). In contrast, the neuroectodermal marker *Pax6* (Figure 2C) and eye-field marker *Rax* (Figure 2D) were highly upregulated at day 9. Upon culturing and differentiating the cells further on the LN523 matrix until day 22, the differentiated retinal cells exhibited two distinct cell clusters expressing developmental-stage-specific retinal transcription markers. The two clusters displayed predominant expression of *Vsx2* and *MITF*, respectively, recapitulating the optic cup phenotype observed *in vivo* (Figures 2E and 2F). Approximately 17% of the retinal cells expressed the photoreceptor progenitor marker *CRX* mRNA as early as on experimental day 32 (Figure 2G–2J) based on H1 hESC photoreceptor differentiation. The CRX-positive cell cluster exhibited diminished transcript expression of *Pax6* and *Vsx2*, suggesting that differentiation toward the photoreceptor lineage was in progress at day 32 (Figures 2I and 2J). In addition, the cells also co-expressed *RCVRN*, rod *Nrl*, and cone *Pde6H* precursors (Figure 2H–2J), indicating that the maturation of photoreceptor progenitors was also underway at day 32. We also observed reduced expression of *MITF* mRNA but increased expression of *CRX* and *NRL* mRNAs at day 32 compared with day 22 (Figures 2I, 2J, and S3), suggesting that the differentiation protocol drove the cells specifically toward the photoreceptor lineage and not RPE. Whole-transcriptome analyses carried out on samples from differentiation days 0, 9, 22, and 32 revealed high reproducibility and little variation in passage-dependent differentiation. We noted a high level of similarity in the overall expression profiles of the photoreceptor differentiation genes, with only 0.1%–8.8% differences (across time) in their expression patterns between passages of H1 hESCs (Figure 2K). Interestingly, basagin (*BSG*) and glucose transporter 1 (*GLUT1*, also known as *SLC2A1*) receptor mRNAs were found to be significantly expressed in differentiated cells from day 9 onward and enriched in the *CRX*-positive cell cluster at day 32 (Figures 2I and 2J). These receptors are known to bind to the trophic factor rod-derived cell viability factor (RdCVF), which is important for the survival of cone photoreceptors.^35^^,^^36^^,^^37^^,^^38^ Taken together, the single-cell transcriptome analyses showed that the temporal expression of the transcription markers for the photoreceptor progenitors recapitulated some of the main steps of retinal development that are observed *in vivo*.^13^

**Figure 3:**
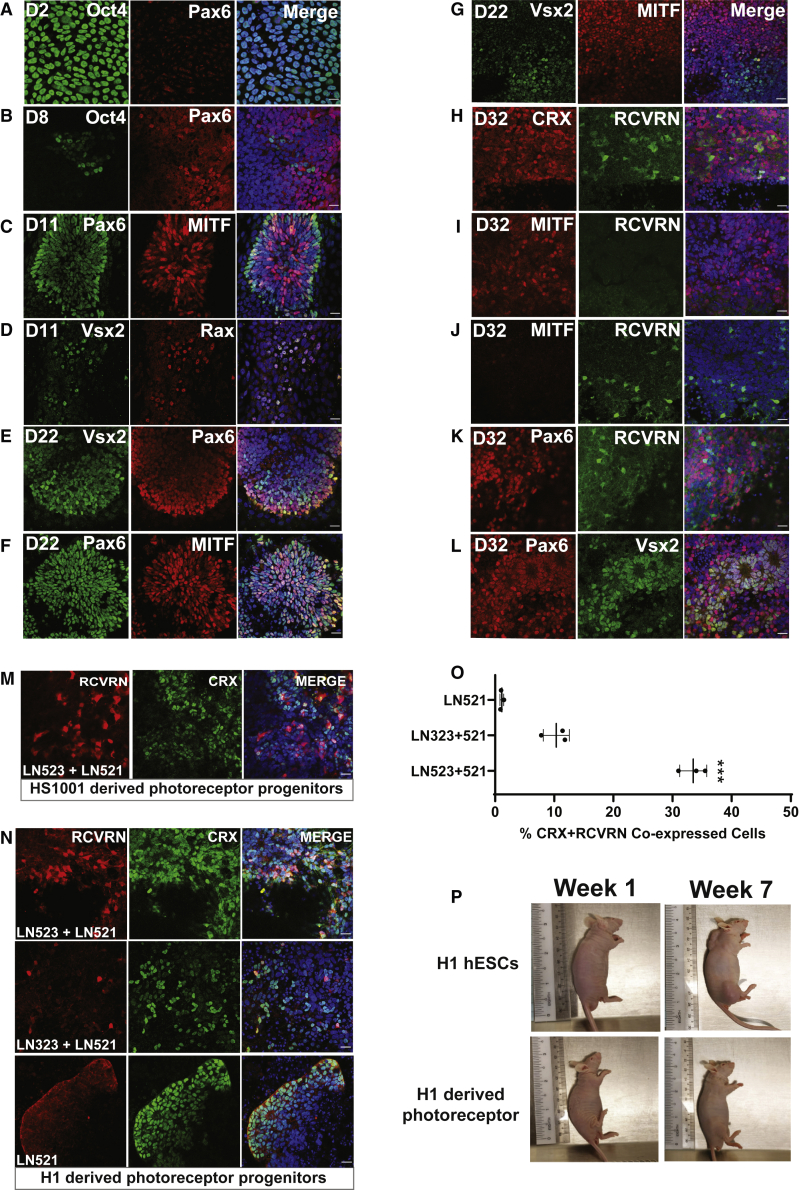
Temporal expression of retinal and photoreceptor progenitor markers and teratoma assay analysis Immunofluorescence analyses of (A) day 2, (B) day 8, (C and D) day 11, (E to G) day 22 and (H to L) day 32 photoreceptor progenitors differentiated from hESCs on retina-specific LN523 + LN521. (M) Reproducibility of LN523 + LN521-based photoreceptor differentiation method in HS1001 hESCs as indicated by co-expression of CRX and RCVRN at day 32 cells. Scale bar, 20 μm. (N) Quantitative analysis showing that approximately 30% of cells differentiated on LN523 + LN521 co-expressed CRX and RCVRN. Scale bar, 20 μm. (O) Higher co-expression of CRX and RCVRN observed in day 32 H1-derived photoreceptors differentiated on LN-523 + LN521 compared with those differentiated on LN323 + LN521 and LN-521. (P) Nude mice developing tumors at 7 weeks post injection with 3 million hESC H1 cells in contrast to absence of tumors in mice injected with day 32 hESC-derived photoreceptor progenitors (n = 3 for each cell type).

Consistent with the temporal transcriptomic analyses results, the expression of pluripotency protein marker Oct3/4 was significantly reduced on day 9 compared with day 0 (Figures 3A and 3B). This further supports a rapid loss in the pluripotency of the hESC lines when they are differentiated on LN521 + LN523 matrix. Furthermore, the increased expression of Pax6, MITF, Vsx2, and Rax on day 11 (Figures 3C and 3D) and day 22 (Figures 3E–3G) suggested that the cells had differentiated toward the optic vesicle developmental stage. The expression of MITF and Vsx2 at day 22 were mutually exclusive (Figure 3G), which suggested that MITF-positive cells could be directed to differentiate into RPE cells, whereas Vsx2-positive cells developed into the neural retina lineage. On day 32, the cells co-expressed CRX and RCVRN, but lacked Pax6 and Vsx2 (Figure 3H–3L) and also displayed significant reduction in MITF expression (Figures 3I and 3J). This suggested that the transplanted day 32 photoreceptor progenitors were en route to specification into mature photoreceptors. The differentiation method based on retina-specific laminins was reproducible in another hESC line, HS1001, where co-expression of CRX and RCVRN was observed at day 32 using immunofluorescence analysis (Figure 3M).

To investigate the efficacy of different retina-specific laminin isoforms in driving the differentiation of pluripotent cells into photoreceptor progenitors, we compared three different retina-specific laminin combinations: LN523 + LN521, LN323 + LN521, and LN521. Based on immunofluorescence analysis at day 32, approximately 33.5% of the cells differentiated on LN523 + LN521 co-expressed CRX and RCVRN (Figures 3N and 3O). Importantly, LN523 + LN521 displayed a higher differentiation efficiency of ∼23.2% and 32.4% compared with LN323 + LN521 and LN521 respectively (Figure 3O). Next, we investigated whether the photoreceptor progenitors derived from hESCs on day 32 had any tumorigenic effects after they were transplanted into nude mice. Based on a tumorigenicity assay that was carried out at 7 weeks after transplantation, there was absence of teratoma formation in nude mice injected with photoreceptor progenitors derived from day 32 hESCs, in contrast to the ones injected with hESCs (Figure 3P). This indicates that our cells differentiated on retina-specific laminins do not risk teratoma formation, thus making them potentially safe for transplantation.

Next, a differential gene expression analysis between day 32 cells differentiated on LN523, LN323, and LN521 matrices identified thousands of genes that were regulated by each laminin isoform (Table S2), suggesting distinct roles of each laminin chain during the generation of photoreceptor progenitors. Compared with LN323, LN523 showed upregulation of Cone-Rod Homeobox (*CRX*), which interacts with recoverin (*RCVRN*) and inter-photoreceptor matrix proteoglycan 2 (*IMPG2*), which are involved in visual and sensory perception of light stimuli (Table S2; Figure S4), an effect potentially induced by laminin α5 chain at day 32 during the photoreceptor progenitor stage. In addition, genes encoding for G-protein-coupled receptors and apical membrane-associated proteins were downregulated in cells differentiated on LN523 compared with LN521, which could be due to the effects of the laminin γ3 chain (Figure S5). These results provide new insights into the functions of specific laminin chains present in the LN523 heterotrimer during photoreceptor specification. Our previous studies that demonstrated LN221-dependent differentiation of hESCs into cardiomyocytes^23^ and LN511-dependent expansion of human epidermal keratinocytes^24^ further validate the specific roles played by different laminin isoforms.

In order to investigate the similarities between our hESC-derived photoreceptor progenitors and human fetal retinal cells, we compared single-cell transcriptomic data and performed pairwise cluster correlations. As shown in Figure S6A, we found that our day 32 hESC-derived photoreceptor progenitors recapitulate day 80 and 82 fetal retinas.^19^^,^^39^^,^^40^^,^^41^^,^^42^ We also observed similar enrichment of visual-related functions in photoreceptors derived from laminin-based differentiation protocol as in fetal retina (Figures S6B and S6C).

Interestingly, we also found that *BSG* and *GLUT1* receptors are expressed at an early stage in the photoreceptor progenitors that are differentiated from hESCs (Figures S7A–S7F). Therefore, we went on to study the effects of exogenous RdCVF on LN523 + LN521-dependent differentiation of photoreceptors. At day 32, we observed a significant increase in the expression of *OPN1MW* (opsin medium wavelength) transcript and protein, compared with *OPN1LW* (opsin long wavelength), in differentiated photoreceptors supplemented with 50–400 ng of RdCVF or RdCVF-FL (Figures S7G and S7H). In contrast, no further increase in efficiency was observed when 800 ng of RdCVF/RdCVF-FL (RdCVF/L) was added. In addition, we performed fluorescence-activated cell sorting (FACS) analysis experiment using DAPI and annexin V to study the cell death between control and RdCV/RdCVF-FL-treated cells. While DAPI staining is excluded from healthy live cells, annexin V staining identifies early detection of apoptotic cells. We found that there are no significant differences in DAPI and annexin V co-stained cells comparing RdCVF/L-treated photoreceptor differentiation with the standard method (Figure S7I). This suggests that there is a possibility that RdCVF/L could have an effect on the specification of cone subtypes.

### Engrafted photoreceptor progenitors improve ERG photoreceptor response in a retina-degenerated rodent model

**Figure 4:**
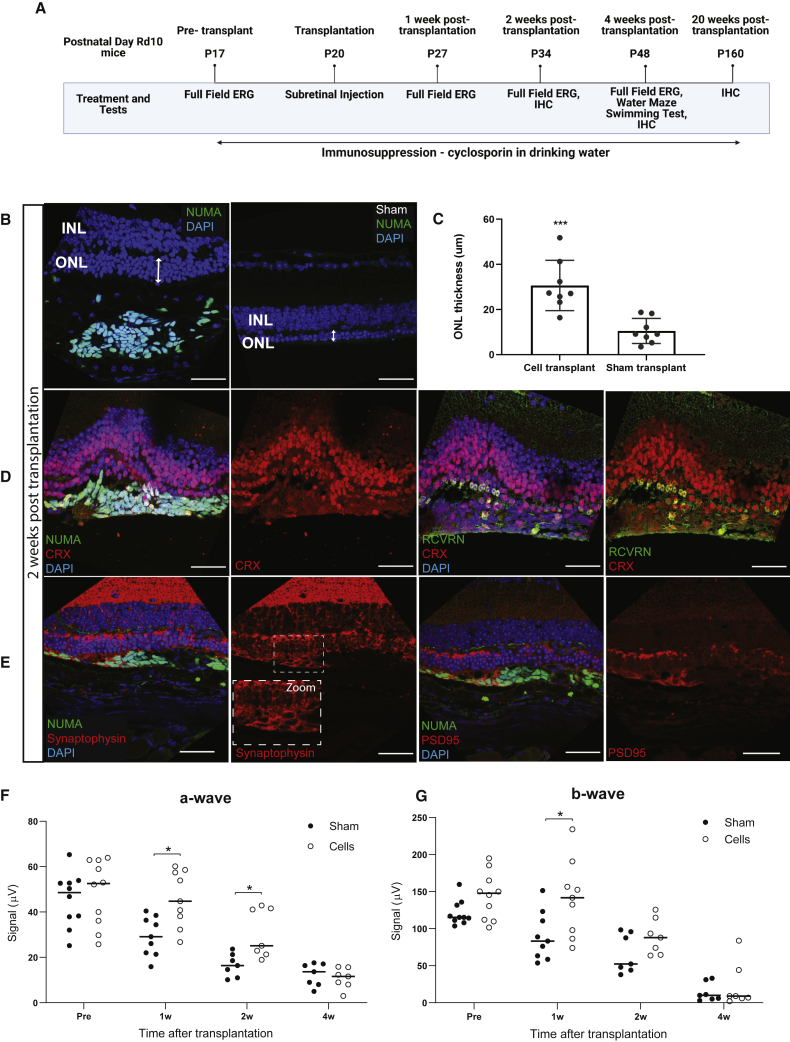
Transplantation and functional analysis in *rd10* mice models of photoreceptor degeneration at 2 weeks post transplantation (A) Schematic timeline of the transplantation and assessment procedures in pre- and post-transplanted *rd10* mice. (B) Representative immunofluorescence analyses show engraftment of differentiated human photoreceptor progenitors (NuMA + human marker) and the preservation of outer nuclear layer (ONL) in transplanted retina compared with sham transplantation (ONL thickness indicated by left-right arrows). (C) Quantification of ONL shows thicker ONL in *rd10* retina engrafted with cell transplants compared with retina engrafted with sham transplants (∗∗∗p <0.005 by ratio paired t test). The differentiated human photoreceptor progenitors engrafted into the mouse retinas (D) co-expressed CRX and RCVRN, and expressed (E) pre-synaptic synaptophysin and post-synaptic connectivity protein PSD95. Scale bar, 40 μm. Amplitudes of the ERG (F) photoreceptor cell response (a-wave) and (G) bipolar cell response (b-wave) recorded in nine *rd10* mice post transplantation. Responses were recorded serially in each animal at postnatal day 27 (P27) and P34. In each animal, ERG responses were recorded simultaneously from eyes injected with photoreceptor progenitor cells (cell transplant) and contralateral control eyes injected with media (sham). ∗p < 0.05 by ratio paired t test. Scale bars, 20 μm.

To test whether our hESC-derived photoreceptor progenitors could improve visual function *in vivo*, we used a retinal degeneration 10 (*rd10*) mouse model exhibiting autosomal human retinitis pigmentosa.^43^^,^^44^^,^^45^ This animal model contains a missense mutation (R560C) in the β subunit of the rod cGMP-phosphodiesterase 6 (*Pde6β*) gene that leads to photoreceptor dysfunction and degeneration from postnatal day 18 (P18) to complete degeneration at P160.^46^^,^^47^^,^^48^ Figure 4A shows the schematic timeline depicting our transplantation and assessment procedures in pre-and post-transplanted *rd10* mice. Transplantation of hESC-derived photoreceptor progenitors to *rd10* mouse retinas by subretinal injection was performed at P20 when photoreceptor degeneration was in progress. The successful transplantation of hESC-derived photoreceptor progenitors was confirmed by immunohistochemical staining of the human-specific nuclei marker NuMA. We found that, in *rd10* mouse eyes transplanted with hESC-derived photoreceptor progenitors, the host’s photoreceptor outer nuclear layer (ONL) was largely preserved at 2 weeks post transplantation (P34), compared with mouse eyes transplanted with sham media (Figure 4B). This is further supported by the quantification of ONL thickness (Figure 4C). We also found that the subpopulation of engrafted NuMA+ cells co-expressed CRX and RCVRN (Figure 4D), and the cells engrafted at close proximity to the host retina, also expressed pre- and post-synaptic connectivity proteins synaptophysin and PSD95, respectively (Figure 4E). However, it is unclear whether prominent synaptic connectivity was established between the engrafted cells and host retina after 2 weeks post transplantation. Nevertheless, these results further validate the potential of engrafting xeno-cells into the host retina for the treatment of retinal disorders.

**Figure 5:**
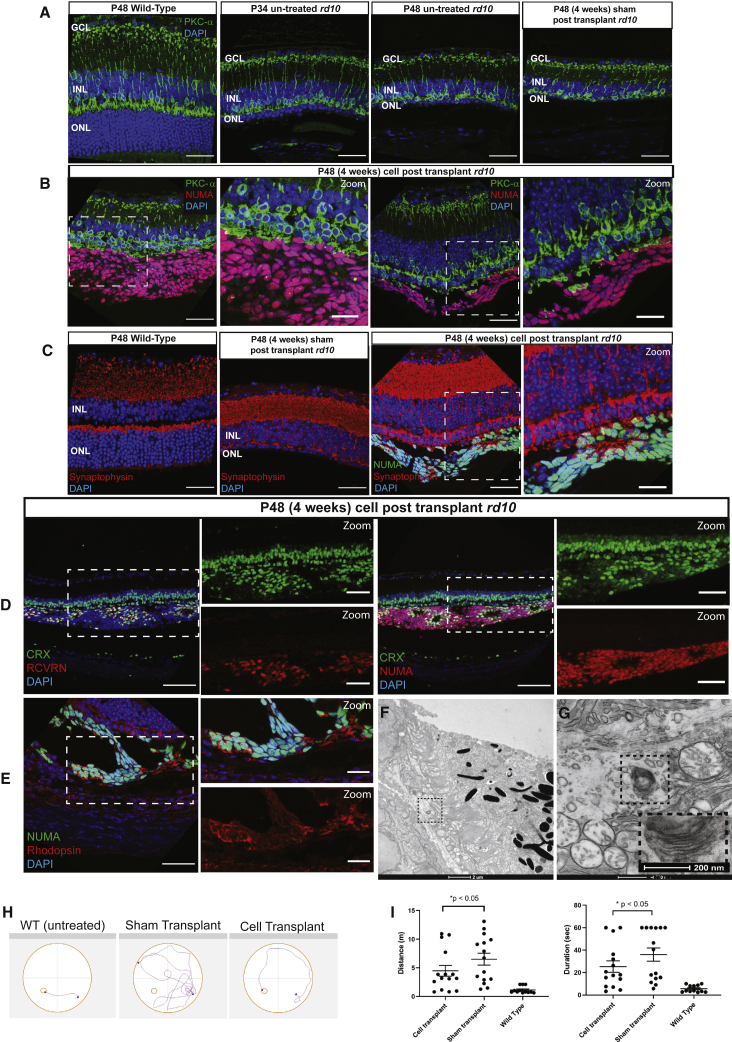
Transplantation and functional analysis in *rd10* mouse model of photoreceptor degeneration at 4 weeks post transplantation. In contrast to (A) sham transplanted *rd10* at P48,(B) the neuronal processes of host protein kinase C (PKC)-α-positive rod bipolar cells extend beyond the host’s INL and the remaining ONL into the closely localized engrafted cells at 4 weeks post transplantation. (C) Pre-synaptic protein synaptophysin was expressed in the subpopulation of transplanted cells and in proximal regions that are connected with the host’s remaining ONL. (D) The differentiated human photoreceptor progenitors (NUMA+) engrafted into the mice showing co-expression of CRX and RCVRN, and the expression of (E) the mature photoreceptor marker, rhodopsin. Scale bars, 40 μm. (F) Transmission scanning electron micrograph showing 2,900× magnification of engrafted hESC-derived photoreceptor progenitors in *rd10* rodent retina. Scale bar, 2 μm. (G) Zoomed-in image of the boxed region in (F) magnified 18,500×, with scale bar representing 500 nm. Inset contains zoomed-in image that is further magnified 30,000×, with scale bar representing 200 nm. (H) Representative swimming patterns in water maze test showing comparisons between wild-type , sham-, and cell-transplanted mice. (I) Statistically significant improvement (∗p < 0.05 by one-way ANOVA) shown in swimming distance and duration for cell-transplanted mice compared with sham-transplanted mice.

To further investigate if there is functional synaptic connectivity readout, we used the full-field flash ERG to assess retinal function serially in *rd10* mice at P27 and P34, corresponding to 7 and 14 days after cell transplantation (Figure 4A). It is important to note that each mouse was tagged individually, and ERG readings were measured longitudinally at the two time points. Amplitudes of the a-wave derived from the photoreceptors (Figure 4F) and the b-wave derived from the bipolar cells (Figure 4G) were significantly higher in mouse eyes engrafted with xeno-cells than in eyes injected with contralateral media at 1 week post transplantation. Most importantly, the photoreceptor-derived a-wave continued to be significantly higher in cell-engrafted eyes than in sham-transplanted eyes. This suggested that the host ONL preservation was conferred by the protective effects of the engrafted cells over a span of 2 weeks after transplantation. This protection was however lost at 4 weeks, where there were no significant differences in a- or b-wave amplitudes between the cell- and sham-transplanted eyes. Interestingly, we found that cell engraftment was still prominent at 4 weeks post transplantation, as marked by the presence of NUMA+ cells (Figures 5B–5E) in P48 *rd10*-transplanted mice. As the host retina deteriorates more progressively with persistent, localized cell engraftment at 4 weeks post transplantation at P48, it is unclear if the full-field ERG is sufficiently sensitive to detect any functional improvement conferred by localized cell engraftment. We started to study whether there is any connectivity between the engrafted cells and the host inner retinal cells. In contrast to wild-type and sham-transplanted eyes (Figure 5A), the neuronal processes of the host rod bipolar cells as indicated by the protein kinase C (PKC)-α expression extended beyond the host’s inner nuclear layer (INL) and the remaining ONL into the closely localized engrafted cells (Figure 5B). The pre-synaptic protein synaptophysin was expressed in the subpopulation of transplanted cells and in the regions that are in close proximity to and connecting with the host’s remaining ONL (Figure 5C). Subpopulation of the engrafted human photoreceptor progenitors (NUMA+) not only co-expressed CRX and RCVRN (Figure 5D) but also contained a subset of cells that expressed the mature photoreceptor marker, rhodopsin (Figure 5E). We then performed transmission electron microscopy (TEM) analysis to determine the presence of any mature photoreceptor structures. Our TEM micrographs identified possible regions where mature photoreceptor disks, which were observed to be small and scarce, could be found in the engrafted maturing hESC-derived photoreceptor progenitors (Figures 5F and 5G). Subsequently, we sought to examine the visual function of the *rd10* mice at 4 weeks post transplantation, using the water maze swimming test. We found that cell-transplanted *rd10* mice showed significant improvement in swimming distance and duration while finding the platform, compared with sham-transplanted mice (Figures 5H and 5I) (∗p < 0.05 by one-way ANOVA): 73% of the cell-transplanted *rd10* mice swam less than 4 m, compared with 33% of the sham-transplanted mice, and 80% of the cell-transplanted *rd10* mice swam for less than 40 s, compared with 47% of the sham-transplanted mice, before they reached the platform. These results suggested that there was visual improvement in cell-transplanted mice, compared with sham-transplanted mice, at 4 weeks post transplantation.

**Figure 6:**
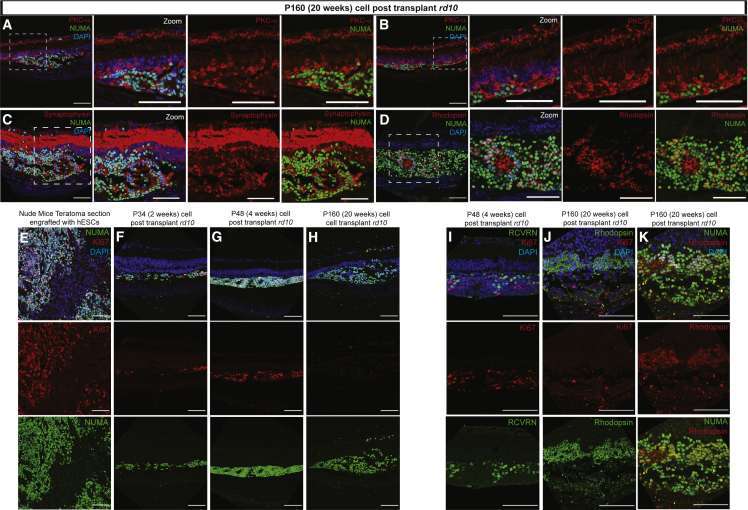
Transplantation analysis in *rd10* mice model of photoreceptor degeneration at 20 weeks post transplantation (A-B) Cell bodies and neuronal processes of PKCα-expressed bipolar cells from the host inner retina layer found associated with the transplanted NUMA-positive cells at 20 weeks post transplantation in the absence of host ONL. (C) Extensive expression of synaptophysin-positive synapses found in surrounding NUMA-positive cells from host INL. (D) Increased number of mature photoreceptor marker, rhodopsin-expressed cells were found in NUMA-positive cells after 20 weeks post transplantation. (E) Positive control shows abundant Ki67-positive cells in teratoma section of engrafted immunosuppressed mice. (F) Presence of proliferative Ki67-positive cells found at 2 weeks and (G) 4 weeks, which is in contrast to (H) 20 weeks post-transplanted eyes. (I) Increased number of mature photoreceptor RCVRN and (J) rhodopsin-expressed cells present at 4 weeks and 20 weeks post transplantation respectively, (K) devoid of colocalization with diminishing Ki67-positive cells. Scale bars, 40 and 80 μm.

Next, we sought to investigate the transplantation outcome at 20 weeks after transplantation at P20 of *rd10* mice. We found that NUMA-positive cells were still present and surviving with the absence of host ONL after 20 weeks post transplantation (Figures 6A and 6B). The cell bodies and neuronal processes of PKCα-expressed bipolar cells from the host inner retina layer were found to be associated with the transplanted NUMA-positive cells (Figure 6A). Additionally, extensive expression of synaptophysin synapses were also found to be established surrounding the NUMA-positive cells, further suggesting increased synaptic connectivity of engrafted cells with the host retina (Figure 6C). Increased distribution of transplanted cells expressing rhodopsin, a mature photoreceptor marker, was observed (Figure 6D). In this long-term cell engraftment model, the engrafted maturing cells have demonstrated the potential to replace the loss of host ONL by cell integration with the association of host bipolar cells and synapses. This is different from the 2-weeks post-transplanted retina, where there could be possibilities of exchange of cellular material or secretory trophic factors to preserve remaining host ONL. Subsequently, we focused on the long-term effects of cell engraftment. We used antibody specific for Ki67, a well-established cell proliferation marker, and Ki67-positive cells were found abundantly in teratoma sections of immunosuppressed mice, as expected in this positive control (Figure 6E). Presence of Ki67-positive proliferative cells was found in 2- and 4-weeks post-transplanted *rd10* retina (Figures 6F and 6G). However, the number of Ki67-positive cells was significantly reduced in 20-weeks post-transplanted eyes (Figure 6H). The diminishing number of Ki67-expressing cells was accompanied by increased number of mature photoreceptor marker, RCVRN and rhodopsin-expressing cells absent of Ki67 expression, in 4- and 20-weeks post-transplanted eye (Figures 6I–6K). This strongly suggested that there is reduced potential of teratoma formation caused by engrafted cells that have undergone *in vivo* photoreceptor maturation and cell integration with the host retina.

### Photoreceptor progenitors differentiated on laminin 523 engrafted in a large-eyed model exhibited potential synaptic connectivity

**Figure 7:**
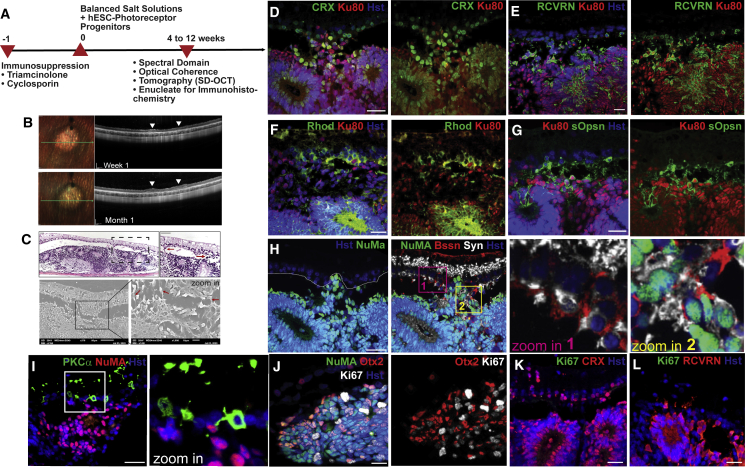
Transplantation method and post-transplantation analyses in rabbit model of induced retina degeneration (A) Schematic depiction of the transplantation procedure in rabbits. (B) Spectral-domain optical coherence tomography (SD-OCT) analysis of the rabbit retina orthotopically transplanted with day 32 hESC-derived photoreceptor progenitors. Arrowheads indicate engrafted hESC-derived photoreceptor progenitors, 1 week after transplantation and at 1-month follow-up. Scale bars, 200 μm. (C) H&E-stained section and scanning electron micrographs confirming the transplantation of hESC-derived photoreceptor progenitors in the rabbit’s subretinal space. The magnified region depicts possible connectivity between engrafted hESC-derived photoreceptor progenitors and the recipient’s retina (see red arrows). Scale bars of 50 and 10 μm, respectively. Immunofluorescence analyses showing engraftment of differentiated human photoreceptor progenitors (Ku80+/NuMA+ human marker) in the rabbit retina. The engrafted cells express photoreceptor progenitor markers (D) CRX, together with cone and rod markers (E) recoverin (RCVRN), (F) rhodopsin (Rhod), and (G) s-opsin (sOpsn). (H) The white dashes mark the boundary between the differentiated human photoreceptor progenitor cells (NuMA/Hoechst positive) and the rabbit’s retina (Hoechst positive; NuMA negative). The pre-synaptic marker Synaptophysin and the active synaptic marker Bassoon are present in the rabbit retina (zoom in 1), and in the integrated hESC-derived photoreceptors progenitors (zoom in 2). (I) Neuronal process extends from host’s PKCα-positive bipolar cell to engrafted NuMA+ cells. The engrafted cells express post-mitotic photoreceptor progenitor markers (J) Otx2, (K) CRX, and (L) RCVRN that do not co-localize with Ki67-positive cells. Scale bars (D–L), 50 μm.

We have demonstrated previously that the injection of saline solution into the subretinal space of rabbits induces retinal degeneration,^49^ while the injection of hESC-derived RPE cells integrates functionally and rescues degeneration of the photoreceptors.^50^ Using the induced rabbit model for photoreceptor degeneration, we studied the *in vivo* functions of day 32 hESC-derived photoreceptor progenitors (Figure 7). The transplanted progenitor cells were found to be present in the subretinal region as early as 7 days post transplantation, where they sustained for over 1 month, devoid of native ONL, as observed by non-invasive *in vivo* spectral-domain optical coherence tomography (SD-OCT) (Figures 7A and 7B). The engraftment of hESC-derived photoreceptor progenitors into the subretinal region was confirmed by histological and scanning electron micrography (SEM) analyses (Figure 7C), which displayed potential connectivity between the engrafted hESC-derived photoreceptor progenitors and the host’s retina cells. Even though histological staining identified the presence of rosettes, which are indicative of premature photoreceptor progenitors, more mature hESC-derived photoreceptors constituted the majority of the transplanted grafts in the rabbit orthotopic xenograft model. The photoreceptor progenitors engrafted into the rabbit retina were confirmed to be derived from hESCs by the presence of human-specific nuclei markers (NuMA or Ku80) with photoreceptor lineage markers CRX and RCVRN (Figures 7D and 7E). In addition, we also detected the presence of rod and cone photoreceptor markers rhodopsin and s-opsin (Figures 7F and 7G), which indicated that our hESC-derived photoreceptor progenitors maintain their retinogenesis profile *in vivo*. Despite the expression of mature photoreceptor markers in the engrafted cells, we did not observe the formation of mature photoreceptor structures in transplanted rabbits, such as photoreceptor disks identified in the *rd10* transplanted mice. Nonetheless, the presence of active synapses between the engrafted human photoreceptor progenitors and the host rabbit retina (Figure 7H, dashed line) was revealed by the expression of the pre-synaptic marker Synaptophysin and the active synaptic marker Bassoon , indicating the formation of ribbon synapses between the graft and host cells.^51^ Specifically, Synaptophysin and Bassoon colocalization was confirmed in the rabbit retina (Figure 7H, zoom in 1; NuMA-negative nuclei) as well as in the engrafted hESC-derived photoreceptors progenitors (Figure 7H, zoom in 2; NuMA-positive nuclei). We also found the neuronal processes of host PKCα-expressed bipolar cells associate with NuMA-positive engrafted cells (Figure 7I), similarly to *rd10* engrafted eyes. These engrafted cells also expressed Otx2, CRX, and RCVRN, which are markers of post-mitotic photoreceptor lineage (Figures 7J–7L),^52^ and showed that they did not co-localize with Ki67-positive proliferative cells, highlighting the post-mitotic status of the day 32 hESC-derived photoreceptor progenitors in the rabbit’s graft.

Taken together, these findings suggest that our day 32 hESC-derived photoreceptor progenitors are integrated and possibly connected with the rabbit host retinal cells. Moreover, these results are congruent with our findings in engrafted *rd10* post-transplanted rodent eyes. The analyses of the cell engraftment are similar in the two animal models. This further underscores the reproducibility of cell transplant outcome in genetic and injury-induced photoreceptor degenerative models that potentially could translate similarly in a human therapeutic context.

## Discussion

The goal of this study was to generate competent photoreceptor cells that can be transplanted into the human host to restore retinal functions, which are lost in diseases such as retinitis pigmentosa and potentially AMD. Here, we have developed a method for the *in vitro* generation of human photoreceptor progenitor cells from pluripotent hESCs by culturing the stem cells in the presence of a highly retina-specific laminin isoform, LN523. Based on extensive transcriptomic and immunohistochemical analyses, we have shown that it is possible to generate hESC-derived photoreceptor progenitors in a highly reproducible manner (Figures 1C, 2K, and 3A–3M). The photoreceptor progenitor markers *CRX*, *RCVRN*, *NRL*, and *PDE6H* were expressed as early as at day 32, while pluripotency markers became strongly downregulated at this time point (Figures 2I and 2J). While 17% of the cells were CRX+ based on single-cell transcriptomic analysis, 33.5% of the cells were CRX+ and RCVRN+ based on immunofluorescence analysis at day 32. Our findings suggest that the retina-specific laminin LN523, in combination with the ubiquitous BM laminin LN521, induces signals that drive the differentiation of hESCs into the photoreceptor progenitor lineage. The two laminin isoforms mediate these effects, likely by mimicking the retina extracellular matrix (ECM) niche that supports early retinogenesis *in vivo*, as demonstrated by the identification of genes that are regulated by specific retina laminin chains (Figures S4 and S5). Similar effects on stem cell differentiation have been described for other cell-type-specific laminin isoforms, including LN221,^23^ LN111,^25^ and LN511,^24^ which are responsible for the specification into cardiomyocytes, dopamine neurons, and keratinocytes, respectively.

Several previous reports have documented inconsistencies in the existing differentiating protocols for the generation of hPSC-derived photoreceptors on cell culture matrices.^10^^,^^11^^,^^12^^,^^13^^,^^14^^,^^15^^,^^16^^,^^17^^,^^18^^,^^19^^,^^41^ Such inconsistencies may be due to batch variations in the animal sera used (e.g., fetal bovine serum) and the cell culture matrix Matrigel extracted from murine Engelbreth-Swarm-Holmes tumor tissue, which contains a large number of extracellular and intracellular mouse proteins.^53^^,^^54^ Thus, these methods may be considered clinically unsafe for the generation of therapeutic cells, posing challenges in their approval for use in human therapies. In contrast, our retinal laminin-based differentiation method does not require manual dissections to separate multiple cell types or re-plating to enrich for neural retina cells.^9^ Therefore, we propose this protocol as a viable and highly reproducible method for the generation of photoreceptors for clinical use.

To assess the viability and reproducibility of our LN523-based differentiation protocol, we have tested it on four other pluripotent hESC lines: HS1001, H9, HS980, and HADC106, and have shown it to be highly reproducible. However, it would be difficult to draw comparisons between our single-cell transcriptomic analyses and other published photoreceptor differentiation protocols due to the many differences that exist between these protocols.

Specifically, our laminin-based differentiation protocol is different from the retinal organoid method, which is highly complex and involves long differentiation durations to generate mature photoreceptors. In support of our experimental findings, the transcriptomic profile of day 32 hESC-derived photoreceptor progenitors has indicated remarkable similarities to that of human fetal retina at developmental stage day 80–82, with enrichment in visual-related functions (Figures S6B and S6C). Even though these progenitors do not express mature photoreceptor markers such as rhodopsin, we postulate that these post-cryopreserved progenitors still retain their regenerative capability after injection into the subretinal space, which is lacking in mature photoreceptors. Indeed, the transplanted day 32 hESC-derived photoreceptor progenitors were observed to express mature photoreceptor marker rhodopsin in 4- and 20-weeks post-transplanted *rd10* mouse eyes, suggesting that the engrafted cells undergo photoreceptor maturation *in vivo*. Intriguingly, the current work shows the presence of RdCVF/RdCVF-FL increases the proportion of the cone subtype, with no significant difference observed in cell death between RdCVF/RdCVF-FL-treated differentiated cells. Although the role of RdCVF in preserving retinal cone survival^35^^,^^36^^,^^37^^,^^38^ (by enhancing aerobic glycolysis through the sequential binding of BSG and GLUT1 receptors)^35^^,^^36^^,^^37^ is well established, we could not exclude the possibility that RdCVF/L is able to mediate this function at different time points of RdCVF/L-treated, laminin-based photoreceptor differentiation. Therefore, the mechanism by which these thioredoxins function in the specification of early human photoreceptor cone subtype remains to be elucidated.

Single-cell transcriptomic studies showed that the photoreceptor marker CRX was more significantly expressed at day 32 compared with day 22 (Figure S3). Hence, day 32 hESC-derived photoreceptor progenitors were used to study transplantation effects in *rd10* mice. We did not perform any cell sorting or purification from the differentiated cell culture to enrich any specific subpopulation. Based on our single-cell transcriptomic analysis of day 32 hESC-derived photoreceptor progenitors, as shown in Figure S3, we do not exclude the possibility that there would be a subpopulation of NUMA+ cells that are non-photoreceptor-specified cells such as ganglion progenitors. Nevertheless, the *in vivo* results presented here for day 32 hESC-derived photoreceptor progenitors have demonstrated significant protective effects on the host ONL, which includes improved ERG a-wave analysis at P34 stage in the *rd10* degeneration rodent model. However, in the current study, we do not exclude the possibility of material transfer between the engrafted hESC-derived photoreceptor progenitor cells and the mouse retina tissues during ONL preservation, as suggested by other reports.^55^^,^^56^^,^^57^ Additional experiments will be required to investigate this possibility. In the rabbit model, the hESC-derived photoreceptor cells transplanted into the host retina were shown to undergo maturation *in vivo* based on the expression of the post-mitotic (Otx2 and CRX) and mature photoreceptor markers (rhodopsin, RCVRN, and opsin). In addition, the expression of the synapse-associated markers (Synaptophysin and Bassoon) in the host retinal region induced to degenerate and, where human photoreceptor progenitors are grafted at 3 months post transplantation suggest that the maturing engrafted photoreceptor cells are able to establish synaptic connectivity with the rabbit host retina tissue. To determine the extent to which these engrafted day 32 hESC-derived photoreceptor progenitors form substantial synaptic connectivity with the host inner retina and exhibit maturation into more photoreceptor cells, we performed long-term studies in the *rd10* rodent model. Our current findings show that at 20 weeks post transplantation, the engrafted maturing cells in the retina are able to replace the loss of host ONL by showing extensive association with the host bipolar cells and synapses. The diminishing number of Ki67-expressing cells was shown to be accompanied by an increased number of mature photoreceptor rhodopsin-expressing cells. These findings strongly suggest the *in vivo* maturation of photoreceptor progenitor cells and their integration with the host’s retina, with reduced potential of teratoma formation. However, it is important to note that the cell transplantation was performed at the P20 stage where there was still remaining ONL, which may help in the long-term engraftment at 20 weeks post transplantation, when the ONL underwent further degeneration.

The current study has certain limitations: first, it lacked a method for purifying photoreceptor progenitors to determine if the long-term retina function could be improved and performing cell transplantation in a fully depleted photoreceptor retina to demonstrate clinical applicability. Second, full-field ERG was only able to detect short-term functional recovery at 2 weeks post transplantation in spite of localized cell engraftment. In order to study the efficacy of the light-evoked host retinal response in longer post-transplanted *rd10* degenerative retinas, full-field ERG may be limited in detecting retinal function, particularly when there are small localized engrafted cell regions. The recently developed microelectroretinograms using MEA could be more sensitive in distinguishing discernible light-evoked spiking activity in retina neuronal cells, as suggested by other reports.^5^^,^^6^^,^^7^ For instance, a recent study by Rebeiro et al. used MEA instead of full-field ERG to measure the targeted light-evoked spiking activity of the enucleated retina transplant that contained the localized cell engraftment site. In the future, it will be important to identify cell surface marker candidates expressed at day 32 that are mediated by human laminin gamma 3 chain. This may enable the sorting of CRX+ photoreceptor progenitor cells, which may potentially lead to significant improvement in the functional retina outcome after transplantation.

In conclusion, this study reveals the role of retina-specific laminin LN523 in driving the differentiation of pluripotent hESCs into the photoreceptor lineage. By studying cell transcriptomic profiles and post-transplanted animal models, we showed absence of teratoma growth and partial improvement in vision, suggesting that the retina-specific laminin-based photoreceptor differentiation method may represent a safe approach for the treatment and management of retinal degenerative diseases. Additionally, the method may also be useful for studying the mechanistic pathways involved in the progression of macular degeneration, thereby leading to the development of alternative therapeutic interventions.

## Materials and methods

### Generation of retina-specific laminin isoforms: LN523 and LN323

#### Collaboration with BioLamina

HEK293 cells were cultured and maintained in basal medium consisting of DMEM-GlutaMAX, high glucose (Life Technologies, 10565042), and supplemented with 10% fetal calf serum (Life Technologies, 10082147) in the presence of Pen-Strep (Life Technologies, 15140122). Before plasmid transfection, HEK293 cells were seeded in the absence of Pen-Strep to help them reach ∼70% confluency at the time of transfection. Human recombinant laminin plasmid (pcDNA3.1) containing human LAMC3 was first transfected into HEK293 cells using Lipofectamine 2000 reagent (Life Technologies, 11668019) and Opti-MEM (Life Technologies, 11058021). Stable, clonal cell lines of HEK003 cells were first established using Hygromycin B (Life Technologies, 10687010) selection with the use of cloning rings (Bel-Art, H37847-0000). This was followed by transfection of LAMB2-containing plasmid into HEK003. HEK023 was subsequently established using Geneticin (G418; Life Technologies, 10131035) and Hygromycin B selections. LAMA5- and LAMA3-containing plasmids were separately transfected into the HEK023 clonal cell line to generate HEK523 and HEK323 clonal cell lines, respectively. The final HEK523/323 cell lines were selected using Zeocin (Life Technologies, R25001), G418, and Hygromycin B.

After selection, the HEK523 and HEK323 cell lines were further expanded and the basal culture medium was switched to serum-free medium for a week. Next, the medium was collected and western blot analysis was performed to detect the presence of LN523 and LN323, which would be secreted by HEK523 and HEK232 cell lines, respectively, and thus confirm the identities of the positive clonal cell lines. The media was dialyzed using dialysis tubing (Spectrum, 132684) with polyethylene glycol (Sigma, 81300) at 4°C for 4–5 days, following which LN523 and LN323 were concentrated in separate Amicon centrifugal tubes (Merck, UFC910096) and purified using the AKTA chromatography system (XK26/100 column, GE Healthcare Life Sciences) and ionic exchange chromatography (MonoQ). Finally, protein fractions were collected and analyzed by Coomassie blue staining and western blotting to identify proteins containing the α5, α3, β2, and γ3 chains present in LN523 and LN323. Mass spectrophotometry was carried out to confirm the identities of retina-specific laminin isoforms in the purified protein fractions.

### Western blotting

Purified protein fraction containing laminins was mixed with 4× LDS NuPAGE sample buffer (Life Technologies, NP0007) and 10× NuPAGE reducing agent (Life Technologies, NP0009). This protein sample was heated at 65°C for 5 min and loaded into 8% NuPAGE Tris-acetate gel (Invitrogen, Thermo Fisher, WG1603). Gel electrophoresis was performed using Novex mini-cell at 120 to 150 V for approximately 1 h at room temperature. After electrophoresis, the protein bands were transferred from the gel to a polyvinylidene fluoride (PVDF) membrane (Bio-Rad, 1620177) in XCell II Blot Module at 30 V for 2 h at 4°C. The PVDF membrane was blocked using 5% blocking buffer (Bio-Rad, 1706404) for 1 h at room temperature and then incubated with primary antibodies overnight at 4°C. The following primary antibodies (1 in 500 dilution in 3% blocking buffer) were used: mouse anti-LAMA5 (Abnova, H00003911-M01), mouse anti-LAMA3 (Atlas, AMAb91123), mouse anti-LAMB2 (R&D Systems, MAB2066), and rabbit anti-LAMC3 (Atlas, HPA022814). The PVDF membrane was then washed in PBS + 0.1% Tween 20 (PBST-20) thrice for 10 min at room temperature. After washing, the PVDF membrane was incubated with secondary antibodies (1 in 2,000 dilution in 3% blocking buffer) overnight at 4°C and washed in PBST-20 thrice for 10 min at room temperature. The protein bands were detected using the ECL Prime western blotting detection reagents (Amersham, GE, RPN2232).

### Immunocytochemistry and imaging

The cells were fixed in 4% paraformaldehyde (Thermo Scientific, 28908) at room temperature for 15 min, following which paraformaldehyde was discarded and the cells were washed in PBS. Next, the cells were permeabilized in blocking solution containing 1× PBS, 0.1% TritonX-100 (PBST), and 5% donkey serum at room temperature for 1 h. Primary antibodies were diluted in fresh blocking solution at 1:200 and incubated with the cells overnight at 4°C. The following primary antibodies were used: mouse anti-Oct3/4 (Santa Cruz, sc-5279), mouse anti-Pax6 (Developmental Studies Hybridoma Bank), sheep anti-Vsx2/Chx10 (ExAlpha, X1180P), mouse anti-MITF (ExAlpha, X2398M), mouse anti-CRX (Abnova, H00001406-M02), sheep anti-Crx (Thermo Fisher, PA5-47897), rabbit anti-RCVRN (Millipore, AB5585), mouse anti-PKC alpha (Thermo Fisher MA1-157), rabbit-OPN1SW (Merck, AB5407), and rabbit anti-OPN1MW (Merck, AB5405). After incubation, the primary antibodies were discarded and the cells were washed in PBST (three 10-min washes) at room temperature. Alexa Fluor 488- and 568-conjugated anti-rabbit/mouse/sheep secondary antibodies (Molecular Probes, A32731, A48286, A11004, A11036, A11015) were diluted in blocking solution at 1:1,000, and were then incubated with the cells overnight at 4°C. DAPI (Sigma, D9542), diluted at 1:500 from stock, was added to the incubation mixture. The stained cells were then washed in PBST (three 10-min washes) at room temperature and ProLong Gold Antifade (Invitrogen, P36930) was added. Image acquisition was performed using LSM710 Carl Zeiss confocal microscope, and ImageJ was used for image analysis, including cell quantification. For cell quantification, three separate images were obtained from three different fields of view using confocal imaging. Cell counter of ImageJ was used to obtain the number of CRX-RCVRN-positive cells and DAPI-positive cells. Percentage of CRX-RCVRN co-expressed cells = (number of CRX-RCVRN-positive cells/number of DAPI-positive cells) × 100%. Average percentage of CRX (green, 488 nm) – RCVRN (red, 568 nm) co-expressed (yellow) cells was obtained based on quantification of three images. This quantification process was repeated for the next two separate batches of photoreceptor progenitor cell differentiation. A t test using GraphPad PRISM was performed to determine whether LN523 + LN521 could result in the highest percentage of co-expressed CRX-RCVRN cells.

### Live FACS analysis

Live cells were trypsinized and co-stained with DAPI (Sigma, D9542) and fluorescein isothiocyanate (FITC) annexin V (BioLegend, #640905), which were diluted in 10% goat serum at 1 in 100. They were incubated at room temperature for 1 h and analyzed using MACQuant Analyzer Flow Cytometer (Miltenyi Biotec).

### Mass spectrometry

#### Preparation of proteins (in gel)

The purified laminin-containing protein fraction was separated on an 8%–20% gradient SDS-PAGE gel and subjected to in-gel digestion.^58^

### Liquid chromatography with tandem mass spectroscopy

The peptides were separated and analyzed using a Dionex Ultimate 3000 RSLCnano system coupled to a Q Exactive instrument (Thermo Fisher Scientific, MA, USA). Separation was performed on a Dionex EASY-Spray 75 μm × 10 cm column packed with PepMap C18 3 μm, 100 Å (Thermo Fisher Scientific), using solvent A (0.1% formic acid) and solvent B (0.1% formic acid in 100% acetonitrile [ACN]) at a flow rate of 300 nL/min with a 60-min gradient. Peptides were then analyzed on a Q Exactive apparatus with an EASY nanospray source (Thermo Fisher Scientific) at an electrospray potential of 1.5 kV. Raw data files were processed and searched using Proteome Discoverer 1.4 (Thermo Fisher Scientific). The Mascot algorithm was then used for data searching to identify proteins. Mass spectroscopy was performed at the Mass Spec Core Facility in the School of Biological Sciences, Nanyang Technological University, Singapore.

### Maintenance of hESC culture

All human pluripotent stem cell studies were carried out in accordance with the National University of Singapore’s Institutional Review Board (IRB 12-451 and N-17-074E). The hESC lines H1 and H9 were from the WiCell Research Institute, WA01 and WA09), HADC106 from the Hadassah Human Embryonic Stem Cell Research Center, and two other cell lines, HS1001 and HS980, were derived at the Karolinska Institute. The hESCs were maintained on culture plates pre-coated with 10 mg/mL of recombinant LN-521 (BioLamina) and fresh NutriStem hPSC XF medium (Satorius, 05-100-1A) was replaced daily.^33^ Cells were passaged at 80%–90% confluence by gentle single-cell dissociation with TrypLE (Gibco, 12563–011) at 37°C for 8 min, followed by centrifugation at 800 rpm for 4 min. Cell pellets was resuspended in NutriStem medium after supernatant was discarded.

### Laminin-based photoreceptor differentiation from hESCs

The hESCs were seeded and propagated *in vitro* on LN521, where they maintained pluripotency.^33^ The hESC line H1 (WiCell Research Institute) was cultured as a monolayer on laminin pre-coated plates and maintained in NutriStem hPSC XF medium. The culture plates (Costar, 3526) were coated with purified human laminin isoforms at 10 μg/mL and incubated overnight at 4°C, according to the manufacturer’s instructions (BioLamina). Upon reaching confluency, the cells were sub-cultured by trypsinization using TrypLE (Gibco, 12563–011) for 8 min at 37°C, 5% CO_2_. The H1 cell line was routinely passaged at 10,000–20,000 cells/cm^2^ in 24-well tissue culture plates (Costar, 3526) coated with LN521 or LN523 + LN521 (2:1) or LN323 + LN521 (2:1), to obtain a final concentration of 10 μg/mL. Following this, the hESCs (H1) were plated at a seeding density of ∼70,000 cells/well and maintained in NutriStem, with the medium changed daily for 2 days. The basal medium used throughout the differentiation process consisted of Glasgow Minimum Essential Medium (GMEM; Gibco, 11710–035) supplemented with 0.1 mM β-mercaptoethanol (Life Technologies, 21985–023), 1× non-essential amino acid solution (Gibco, 11140–050), and 1 mM pyruvate (Gibco, 11360–070). After reaching about 70% confluency in 2 days, the NutriStem was replaced with neuroectodermal induction medium (NIM). NIM consisted of 2% B27 supplement without vitamin A (Life Technologies, 12587001), 1% N2 supplement (CTS, Life Technologies, A13707-01), 5 μM SB431542 (Sigma, S4317), and 5 μM CKI-7 (Sigma, C0742). NIM was changed every 2 days from day 2 to day 8. On day 9 of differentiation, NIM was replaced with photoreceptor differentiation medium (PRDM) that consisted of 2% B27 supplement without vitamin A, 1% N2 supplement, 10 ng/mL human brain-derived neurotrophic factor (BDNF; peprotech, 450-02-50), 10 ng/mL human ciliary neurotrophic factor (CNTF; Prospec-Tany Technogene, CYT-272), 0.5 μM retinoic acid (Tocris Bioscience, 0695/50), and 10 μM N-[N-(3,5-difluorophenacetyl-L-alanyl)]-S-phenylglycine t-butyl ester (DAPT; Selleckchem, S2215). This medium was changed every other day until D32.

### Cryopreservation of hESC-derived photoreceptor progenitors

Day 32 hESC-derived photoreceptor progenitors were dissociated with TrypLE at 37°C for 8 min, followed by centrifugation at 800 rpm for 4 min. The cell pellet was resuspended in 1 mL of serum-free cryopreservation medium mFreSR (STEMCELL Technologies, 05854) after supernatant was discarded. Each cryovial contained approximately 2 × 10^6^ cells/mL and was stored in liquid nitrogen until transplantation.

### Quantitative PCR analysis

Total RNA from hESC-derived photoreceptor progenitor cells was extracted and purified using RNeasy Mini Kit (Qiagen, 74104), according to the manufacturer’s instructions. RNA yield and purity were determined using NanoDrop ND-2000 spectrophotometer (NanoDrop Technologies). For quantitative reverse transcription PCR (RT-PCR) analysis, cDNA was synthesized from 1 μg of total RNA using the TaqMan Reverse Transcription Reagents Kit (Applied BioSystems, N8080234) or iScript Kit (Bio-Rad, 1708891), according to the manufacturer’s instructions. Real-time quantitative RT-PCR was performed in assay mix containing iQ SYBR Green Super mix (Bio-Rad, 1708882), primers for genes of interest, and the synthesized cDNA. The primer sequences used are listed in Table S3.^59^

### Single-cell RNA-seq

The Chromium single-cell 3′ reagent kit (10x Genomics) was used to generate Illumina-ready sequencing libraries. We followed the manufacturer’s instructions to generate single-cell 3′ library for day 2, day 9, day 22, and day 32 cells.

### Time-series single-cell RNA profiling of photoreceptor progenitor differentiation

We generated a time-series single-cell RNA-seq dataset for photoreceptor progenitor cells derived from H1 embryonic stem cells using the laminin protocol (LN521 + LN532; Figure 1) at days 9, 22, and 32. We conducted this analysis twice, starting from two different H1 passages. In total, we generated eight 10x libraries (one for each time point and passage). For this, RNA was isolated using the 10x genomics kit and sequenced using the Illumina Hi-Seq3000 sequencing platform by multiplexing the eight samples in eight lanes. Reads were mapped to the human genome (Ensembl version 90) and quantified using Cell Ranger 2.1.1 10x Genomics software. We provided the Cell Ranger with a custom-built reference transcriptome generated by filtering the Ensembl transcriptome for the gene biotypes: protein coding, lincRNA, and antisense. The Cell Ranger was run with the expected number of cells parameter (expect-cells) set to 3000. It estimated 3926, 3274, 4368, 3761, 1790, 4934, 3881, and 1938 cells for D2_P1, D2_P2, D9_P1, D9_P2, D22_P1, D22_P2, D32_P1, and D32_P2, respectively. In all cases, the percentage of reads that could be mapped confidently to the human genome was higher than 93%. The output matrices (i.e., genes.tsv and barcodes.tsv) were then input into R, and genes with zero counts in all cells were discarded.

Next, we carried out independent quality control tests in each 10x library. We removed (1) cells with very low and very high library sizes (i.e., cells below and above the fifth and 99^th^ percentiles of the total cell library sizes, respectively), (2) cells with low number of detected genes (i.e., cells below the fifth percentile of the total gene distribution detected in each cell), and (3) cells with more than 10% of their total gene count coming from mitochondrial genes. In addition, we carried out five gene quality control steps in each independent analysis: (1) we took into account only the detectable genes, which are defined as genes that could be detected with more than one transcript in at least 1% of the total cells; (2) we removed genes with low average expression in the data (i.e., genes with an average expression below 0.01; this cutoff was set based on the total distribution of average gene expression across all cells and all genes); (3) we removed genes with a high dropout rate using the M3Drop 3.09 R package,^60^ which ranked all the genes between low and high false discovery rates (FDRs) and removed the bottom 25% of the genes (i.e., genes with highest dropouts); (4) we removed outlier genes in the gene expression distributions (eg., MALAT1 gene); and (5) we removed genes encoded by the mitochondrial genome. We also normalized gene counts with the scran 1.8.4 R package.^61^ Scran sizes were computed from cell pools by pre-clustering the data with the quickCluster function. The output object of this function was provided to the computeSumFactors function, following which Log2-transformed normalized counts were computed using the normalize function in the scater 1.8.4 R package (using default parameters).^62^ Then, to account for cell cycle effects, we computed the G2M and G1 cell cycle phase scores for each cell by using the cyclone function in the scater 1.8.4 R package, to which we input the set of human cell cycle genes provided in Scialdone et al.^63^ Only the cell and gene sets (across the eight 10x libraries) that passed the quality control steps were considered for further analyses using the Seurat V3 pipeline.^64^ We carried out two separate analyses, one with all the eight samples and one with only day 22 and day 32 (a total of four) samples. UMI counts were normalized by regularized negative binomial regression by using the SCTransform function with default number of variable genes. In this function, we provided the cell cycle scores previously computed for the G2M and G2 phases to regress out these effects. The RunPCA computed 50 principal components by considering just the variable genes. Next, the findElbow function from the ChemoSpecMarkeR R package was run to estimate the number of principal components that could be used for Uniform Manifold Approximation and Projection (UMAP) by using the elbow method. UMAP was run using the RunUMAP (Seurat v3) function, wherein the Seurat package function FeaturePlot was used to color the expression level of marker genes. The FindMarkers function in the Seurat package was used to calculate the differentially expressed (DE) genes between two passages based on the criteria Bonferroni <0.05, gene expressed in >10% cells, and log2 fold change >0.25 or < −0.25. The proportion of DE genes was calculated as the number of DE genes divided by the total number of expressed genes (>10% cells) at day 2, day 9, day 22, and day32. Seurat function DotPlot was used to illustrate how marker genes were altered across different cell clusters. The size of the dot represented the percentage of cells expressing the marker gene within a cell cluster, while the color represented the average expression of the marker gene across all cells within a cell cluster. To validate the reproducibility of our photoreceptor differentiation protocol, we generated single-cell RNA-seq datasets from H1, H9, HS980, and HADC106 embryonic stem cells at days 9 and 32. RNA from these cells was isolated using the 10x genomics kit and sequenced using the Illumina Hi-Seq3000 sequencing platform. Reads were mapped using Cell Ranger and analyzed using Seurat as described above (materials and methods: time-series single-cell RNA profiling of photoreceptor progenitor differentiation). For each cell line (H1, H9, HS980, HADC106), the mean expression level of each gene across all the cells at day 32 was generated from individual Seurat objects. Finally, we computed pairwise correlations of cell lines using Spearman’s ranked correlation by the heatscatter function from the *LSD* R package.

### Bulk RNA-seq

RNA-seq libraries were constructed from total RNA using the Illumina Truseq Stranded Total RNA library preparation kit (with Ribozero Gold) and sequenced on an Illumina HiSeq 3000. Reads were pair-ended to generate 150-bp-long fragments. RNA-seq reads were assessed for quality, aligned to GRCh38.79 using STAR 2.5.2b,^65^ and quantified with featureCounts v1.5.1.^66^ Ribosomal genes (Ensembl gene biotype rRNA) and mitochondrial genes were removed. Gene counts were rounded using the R function “round” and differential expression analysis was performed using DESeq2 1.14.1^67^ with a pre-filtering step, in which only genes with more than one count when summing up across all samples were considered. An LRT test was carried out to compare across time points. Differential expression analysis between time points and conditions was carried out by Wald test implemented by DESeq2 pairwise comparison, with the outlier correction parameter cooksCutoff set to false (and the rest of the parameters set to default). In the DESeq2 model, we added sequencing lanes as covariates and corrected by using DESeq2 reduced model (nbinomLRT function with parameters full = ∼lane + laminin_type, reduced = ∼lane). DE genes with FDR <0.05, calculated by DEseq2 pairwise comparisons, were used for further downstream functional analysis.

### Gene ontology

The involvement of DE genes in biological pathways were tested using the Kyoto Encyclopedia of Genes and Genomes (KEGG), Wikipathway, and Gene Ontology databases by R package clusterProfiler v3.14.3.^68^ Genes involved in the functional pathways were then tested by protein-protein interaction (PPI) network using String-db v11.0.^69^

### Single-cell RNA profiling of published fetal retina

The 10X single-cell transcriptomics data of fetal retina (central and peripheral retina, spanning days 59, 80, 82, 105, and 125) were obtained from Sridhar et al., (GSE142526).39 The Cell Ranger counts were downloaded for fetal retina. Downloaded data, along with the CRX-enriched photoreceptor progenitors differentiated from LN523 + LN521, were individually analyzed using Seurat as described above (section “time-series single-cell RNA profiling of photoreceptor progenitor differentiation”).

### Pairwise cluster correlations

Pairwise correlations between cell clusters across datasets was performed by computing the gene specificity matrices as described by Tosches et al.^70^ The gene specificity matrices were generated by identifying the common genes across clusters. For each pair of clusters across datasets, the specificity score for each common gene was calculated as the ratio of gene expression within the cluster over the global gene expression. The Spearman rank correlation of gene specificity scores was used to compute the correlated clusters across datasets. The correlation heatmap was plotted using pheatmap R package (https://cran.r-project.org/web/packages/pheatmap/pheatmap.pdf).

### Transplantation into rabbits

All animal protocols were approval by Stockholm’s Committee for Ethical Animal Research, and experiments followed the Statement for the Use of Animals in Ophthalmic and Vision Research. A total of six New Zealand white albino rabbits, acquired from Lidköpings rabbit farm (Lidköping, Sweden), were included in the study. The average age of rabbits was 6 months and they weighed 3.5 to 4.0 kg. The rabbits were kept in a 12-h light/dark cycle, with water, hay, and straw supplied *ad libitum*, and dry pellets fed once a day. They were then anesthetized by intramuscular administration of 35 mg/kg ketamine (Ketaminol, 100 mg/mL, Intervet) and 5 mg/kg xylazine (Rompun vet. 20 mg/mL, Bayer Animal Health), and their pupils were dilated with a mix of 0.75% cyclopentolate/2.5% phenylephrine (APL). One week prior to transplantation, the rabbits received an immunosuppression regimen of 2 mg of bilateral intravitreal triamcinolone (Triescence, Alcon Nordic) and systemic cyclosporine-A (20 mg/kg/d by mouth; Teva Sweden AB). They were then culled by intravenous injection of 100 mg/kg pentobarbital (Allfatal vet. 100 mg/mL, Omnidea), and their eyes were enucleated and fixed in 4% buffered formaldehyde (FA; Solveco AB), both free-floating and by intravitreal injection of fixing solution, for histology analyses.

For subretinal transplantations, single-cell suspensions of hESC-derived photoreceptor progenitors obtained from cryostocks were centrifuged at 1,200 × *g* for 8 min, washed twice with PBS, resuspended in sterile PBS at a concentration of 5 × 10^5^ cells/50 μL, and kept on ice until surgery. All the rabbits were then subretinally injected with 50 μL of hESC-derived photoreceptor progenitor cell suspension in both eyes by a two-port transvitreal *pars plana* technique (Alcon Accurus, Alcon Nordic), as previously described.^49^^,^^71^ In PBS-injected rabbits, the host photoreceptors displayed initial signs of degeneration form day 1 post injection and complete loss of ONL at approximately 14 days after subretinal injections, as observed by SD-OCT.^49^ In accordance with the protocols, post-surgical topical steroids or antibiotics were given if needed. Post-transplantation follow-ups were performed by SD-OCT on anesthetized rabbits using a Spectralis HRA+OCT device (Heidelberg Engineering) equipped with the Heidelberg Eye Explorer Software, as previously described.^50^ Briefly, cross-sectional OCT scans were obtained with simultaneous multicolor confocal scanning laser ophthalmoscope (MC-cSLO) reflectance reference images representing the transplanted area. Tissue sectioning and immunostaining for histology were performed using paraffin-embedded eyes, which were serial sectioned into 4-μm layers. The slides were deparaffinized using classic xylene/alcohol/dH_2_O/Tris-buffered saline (TBS, pH 7.6) protocols, and sections of engrafted hESC-derived photoreceptor progenitors were identified by hematoxylin and eosin staining. The expression of specific markers was determined by IHF of sections of interest, in a Bond III robotic system (Leica Biosystems, Newcastle, UK). User-based modifications were made in the antigen retrieval method (EDTA buffer, pH > 8, 20 min, 100°C; Leica Biosystems), primary antibodies used (all 60-min incubation: 1:400 anti-RPE65, Abcam ab78036; 1:100 anti-NuMA, Abcam ab84680; 1:50 anti-Otx2, R&D Systems AF1979-SP; 1:100 anti-Ki67, Dako M7240; 1:50 anti-CRX, Abcam ab140603; 1:500 anti-rhodopsin, Abcam ab221664; 1:500 anti-Ku80, Thermo Fisher MA5-15873; 1:50 anti-recoverin, Thermo Fisher MA1-932; 1:50 anti-synaptophysin, NCL NCL-L-SYNAP-299; 1:50 anti-Bssn, Novus NBP1-46351; 1:100 anti-S-opsin; Sigma-Aldrich A4886, 1:50 anti-PKCα, Thermo Fisher MA1-157) and secondary antibodies used (all 15-min incubation at 1:2,000 from Invitrogen: donkey anti-mouse-Alexa488, A32766; donkey anti-rabbit-Alexa555, A32795; donkey anti-goat-Alexa555, A21432. From Sigma-Aldrich: donkey anti-goat-CF647, SAB4600175). Hoechst 33258 (1:5,000, Sigma-Aldrich H94403) was added to the solution containing secondary antibodies for counterstaining. Images were acquired on an Axioskop 2 plus fluorescence microscope with the AxioVision software (Zeiss, Gottingen, Germany).

### Subretinal injection and electroretinography for *rd10*^*−/−*^ rodents

All the procedures involving animal handling were performed with prior approval and in accordance with the National University of Singapore’s Institutional Animal Care and Use Committee (IACUC) protocols and guidelines for Ethical Animal Research. B6.CXB1-*Pde6b*^*rd10*^/J mice were purchased from Jackson Laboratory. The pups were immunosuppressed from P17 to P30 by feeding them drinking water containing cyclosporine (260 g/L). The P20 pups were anaesthetized with a combination of ketamine and xylazine (20 mg/kg body weight; 2 mg/kg body weight). Their eyes were dilated using a drop each of 1% tropicamide (Alcon Laboratories, Tampa, FL) and 2.5% phenylephrine (Alcon Laboratories, Tampa, FL) ophthalmic solutions. The mice were then placed on a warm pad with their eyes looking straight upward under the surgical microscope (Zeiss Opmi Lumera 700). A scleral incision was made using a sharp 30G needle at the pars plana region. Subretinal injections of 2 μL of hESC-derived photoreceptor progenitor cells (300,000 cells) were given at the opposite side of the retina via a 33G blunt needle inserted through the scleral incision and across the vitreous. Sham injection consisted of only 2 μL of freshly prepared PRDM medium. Successful injections were confirmed by observing the formation of retinal blebs. Retinal function was assessed in mice using the full-field flash ERG at 7, 14, and 28 days after cell transplantation, corresponding to P27, P34, and P48. After 12 h of dark adaptation, animals were anaesthetized with intraperitoneal injection of ketamine (60 mg/kg) and xylazine (10 mg/kg). Pupil dilation and corneal anesthesia were achieved by topical application of one drop of tropicamide (0.5%), phenylephrine (2.5%), and proxymetacaine hydrochloride (0.5%). Next, the animals were placed on a heated stage where their body temperatures were maintained at 37°C ± 0.5°C. Retinal responses to a series of stimuli of varying intensities (0.003 to 100 log cd.s/m^2^) were recorded bilaterally using a Diagnosys Celeris ERG system (Diagnosys LLC, Lowell, MA). Two components of the ERG waveform were analyzed: the P3 response (a-wave), which reflects the activity of the photoreceptors, and the P2 response (b-wave), which reflects the activity of the ON-bipolar cells. The maximum amplitudes of the a-wave were calculated using a delayed Gaussian function and the peak b-wave amplitudes were calculated using a hyperbolic, Michaelis-Menten function.^72^

### Teratoma formation assay

Male CrTac:NCr-Foxn1^nu^ nude mice (8–10 weeks old, 20–25 g) were purchased from InVivos, Singapore, mouse stocks of different genetic backgrounds are available, including both BALB/c inbred and NIH(S) outbred stocks. hESCs or day 32 hESC-derived photoreceptor progenitors (approximately 3 million cells) were resuspended in 30 μL of Matrigel (Corning, 354277) and each aliquot was injected intramuscularly into the hind limb muscle and subcutaneously. The injected mice were then monitored for 7 weeks. Samples of injected tissue were excised, fixed in formalin, and routinely processed through paraffin embedding and staining with hematoxylin and eosin. Images were examined under light microscopy by a certified veterinary pathologist.

### SEM and TEM: sample preparation and imaging

#### SEM

Glass slides with engrafted mouse or rabbit retina sections were de-waxed and immediately coated with a metal layer for SEM imaging. The coating was performed in a JEOL Auto Fine Coater using Au target as the conductive film. At first, all the slides were coated with a thin layer of Au (less than 10 nm) to protect them from ambient conditions when SEM imaging is performed. The process parameters for this first coating were 20 mA sputtering current and 30 s of coating time. Following this first coating, the slides were stored back in their containers until SEM imaging could be performed. Immediately prior to imaging, each sample was fixed to the SEM sample holder using a carbon adhesive film and then a second coating of Au was applied to ensure good electrical conductivity. The final thickness of the conductive coating was estimated to be about 20 nm. Imaging was performed in a JEOL JSM 6010LV scanning electron microscope, equipped with a thermal electron gun. Micrographs were collected using a secondary electron detector and the gun was set at an acceleration voltage of 20 kV.

#### TEM

The lower portion of the posterior segment of the enucleated eyes was fixed in a mixture of 2.5 % glutaraldehyde (EM Sciences, Hatfield, PA, USA) and 2% paraformaldehyde (PFA) (Sigma-Aldrich, St. Louis, MO) in 1× PBS for 2 h at 4°C, and rinsed in 1× PBS three times. Rinsed samples were post-fixed in 1% aqueous osmium tetroxide (OsO_4_, Electron Microscope Sciences), dehydrated in increasing concentrations of ethanol, cleared in acetone, and processed for Araldite resin infiltration and embedding. Semi-thin sections (0.1 μm thick) were cut using a Leica-Reichert Ultracut E ultramicrotome (Leica Biosystems Richmond). The sections were collected on POLYSINE microscope glass slides and stained in a solution of 1% toluidine blue O (Sigma-Aldrich, St. Louis, MO) and 1% sodium borate (Sigma-Aldrich, St. Louis, MO) dissolved in distilled water. The solution was filtered with a hydrophobic membrane filter of pore size 25 μm prior to staining for light microscopy. Ultra-thin sections (70 nm thick) were collected onto a formvar carbon-coated 2 × 1 mm slot grid (Electron Microscope Science, Hatfield, PA, USA) and contrast stained with lead citrate. Imaging was performed using a TEM (Tecnai Spirit G2 120 kV, FEI Company).

### Water maze swimming test

The water maze test is performed based on a protocol^73^ established by the neuroscientist Richard G. Morris in 1981. A circular pool (diameter 150 cm) made of metal was placed in a quiet room. A cylindrical platform/island (height 31 cm) painted black was placed in the pool, and water was filled up until 1 cm of the platform was exposed above the water level. The test consisted of four training and one test session. The training sessions were done under normal lighting conditions, while the test was done under dim-light conditions. The ANY-maze video tracking system (Stoelting, Illinois) was used to record parameters such as latency to enter the platform and to track plots, distance travelled, and average velocity to reach the platform. The pool was divided into four quadrants—NE, NW, SE, and SW—in the software, and the location of the platform was marked by a circle. When the test subject entered the circle, the software stopped the tracking and recorded the time spent to reach the platform. The platform was placed in a random quadrant during each training session. Three training blocks, each consisting of three trial runs, were performed during a single training session, with the mice placed at different starting quadrants. The mice were allowed to swim freely for 60 s in the water to identify the platform. The mice that failed to find the platform were guided to it manually and allowed to rest there for 20 s. Each mouse was towel dried and returned to its home cage after each run. It was also allowed to rest for around 10–15 min between trials. During the test session on day 5, the mice were placed in the water maze under dim-light conditions and allowed to swim freely for 60 s to test their ability to find the visible platform.

#### Synthesis of recombinant RdCVF and RdCVFL

Recombinant proteins were prepared as described previously.^74^ DNA fragments of human RdCVF and RdCVFL were synthesized by Integrated DNA Technologies (IDT). The DNA fragments were inserted into pD441 plasmid at the EcoR1 and HindIII restriction sites, and the His-tagged SUMO tag (H6SUMO) was incorporated into the N-terminal ends of the target proteins. Then the plasmids were transformed into *Escherichia coli* Turbo strain and gene expression was induced by IPTG. After overnight culturing, cells were pelleted and lysed. Tagged proteins were purified using high-pressure liquid chromatography (HPLC) equipped with a 5-mL HisTrap FF column (AKTA Explorer 10 FPLC, GE Healthcare). The tag was cleaved by adding 15 μg/mL His-tagged ULP1 (SUMO protease), and the cleaved tag and ULP1 were removed by passing through the HisTrap column.

## Data and materials availability

We have uploaded the raw transcriptional data (single cell and bulk) to Gene Expression Omnibus (GEO) with accession number GSE161417.

## Supplemental information

Document S1. Figures S1–S7 and Table S3

Table S1. Mass spectrophotometry analysis on the identities of the retina-specific laminin isoform 523

Table S2. Differential gene expression analysis between different photoreceptor progenitors differentiated on LN523, LN323, and LN521 culture matrices at day 22 and day 32

Document S2. Article plus supplemental information
