# Supplementary material for: Photoreceptor laminin drives differentiation of human pluripotent stem cells to photoreceptor progenitors that partially restore retina function
Source: Mol Ther. 2023 Jan 12;31(3):825–46. doi: 10.1016/j.ymthe.2022.12.012 (PMC10014235; doi:10.1016/j.ymthe.2022.12.012)
Supplement: Document S1. Figures S1–S7 and Table S3 [file mmc1.pdf]

YMTHE, Volume *31*

Supplemental Information

Photoreceptor laminin drives differentiation of human pluripotent stem cells to photoreceptor progenitors that partially restore retina function

Hwee Goon Tay, Helder Andre, Vicki Chrysostomou, Swarnaseetha Adusumalli, Jing Guo, Xiaoyuan Ren, Wei Sheng Tan, Jia En Tor, Aida Moreno-Moral, Flavia Plastino, Hammurabi Bartuma, Zuhua Cai, Sai Bo Bo Tun, Veluchamy Amutha Barathi, Gavin Tan Siew Wei, Gianluca Grenci, Li Yen Chong, Arne Holmgren, Anders Kvanta, Crowston Jonathan Guy, Enrico Petretto, and Karl Tryggvason

Table S1. Mass spectrophotometry analysis on the identities of the retina-specific laminin isoform 523.

Table S2. Differential gene expression analysis between different photoreceptor progenitors differentiated on LN523, LN323 and LN521 culture matrices at Day 22 and Day 32.

Table S3. Primer list.

**Primers**	**Forward (5' to 3')**
Long Wavelength Opsin	GCCTACTTTGCCAAAAGTGC
Short Wavelength Opsin	TGTGCCTCTCTCCCTCATCT
Medium Wavelength Opsin	CATCTTTGGTTGGAGCAGGTACT

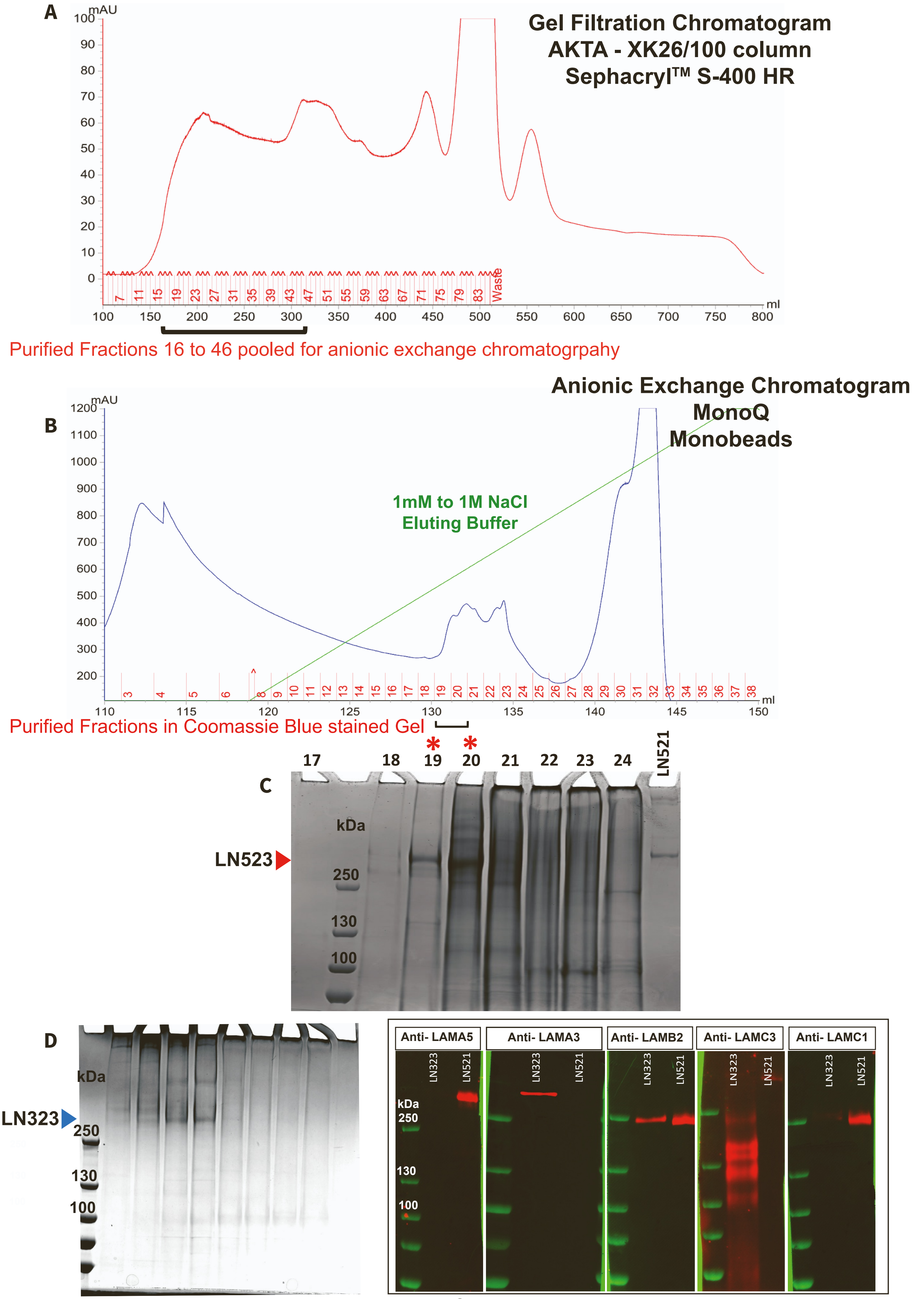
A
Gel Filtration Chromatogram
AKTA - XK26/100 column
SephacrylTM S-400 HR
mAU
ml
Waste
Purified Fractions 16 to 46 pooled for anionic exchange chromatogrpahy
B
Anionic Exchange Chromatogram
MonoQ
Monobeads
1mM to 1M NaCl
Eluting Buffer
mAU
ml
Purified Fractions in Coomassie Blue stained Gel
C
17
18
19
20
21
22
23
24
LN521
kDa
LN523
250
130
100
D
kDa
LN323
250
130
100
Anti- LAMA5
Anti- LAMA3
Anti- LAMB2
Anti- LAMC3
Anti- LAMC1
LN323
LN521
kDa
250
130
100

Figure S1

Figure S1. Purification and analysis of LN523 from HEK293 cell culture media. (A) Gel filtration of dialyzed medium on Sephacryl S-400 HR column. Fractions 15-50 containing high molecular weight proteins in the void volume were pooled. **(B)** Anionic exchange chromatography fractions 17-24 released from the column in the 1mM-1M NaCl gradient (green line) were analyzed on SDS-PAGE gels and laminin size proteins were found in fractions 19 and 20. **(C)** Fractions 19 and 20 were shown to contain laminin 523 and a minor amount of LN521 component chains by western blotting.

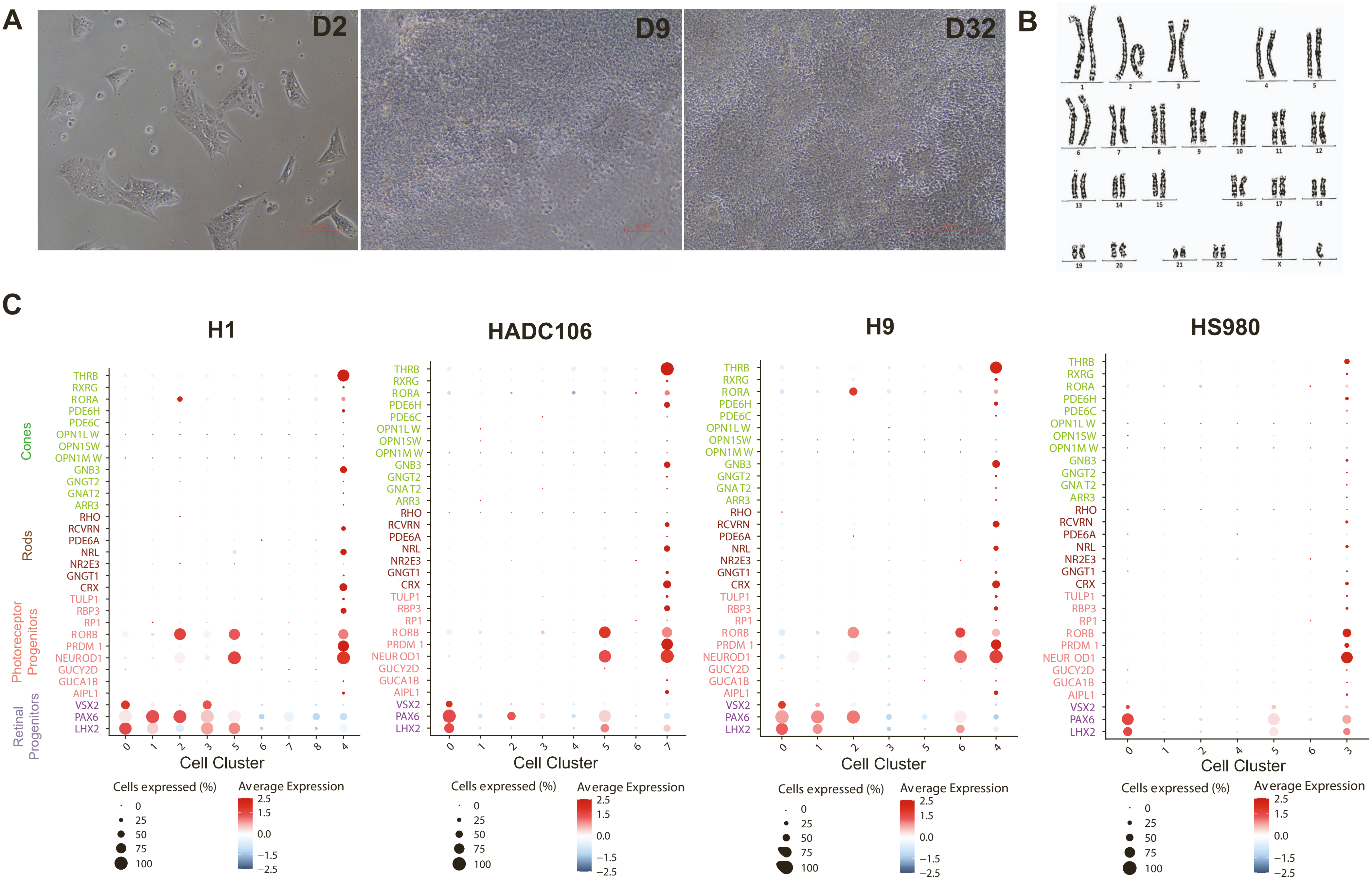
A
D2
D9
D32
B
C
H1
HADC106
H9
HS980
Cones
Rods
Photoreceptor Progenitors
Retinal Progenitors
THRB
RXRG
RORA
PDE6H
PDE6C
OPN1LW
OPN1SW
OPN1MW
GNB3
GNGT2
GNAT2
ARR3
RHO
RCVRN
PDE6A
NRL
NR2E3
GNGT1
CRX
TULP1
RBP3
RP1
RORB
PRDM1
NEUROD1
GUCY2D
GUCA1B
AIPL1
VSX2
PAX6
LHX2
Cell Cluster
Cells expressed (%)
Average Expression

Figure S2

Figure S2. (A) Bright Field images showing Day 2 undifferentiated hESCs and Day 9 and Day 32 hESC-derived photoreceptor progenitors. Scale bar: 100 μm. **(B)** Karyotype analysis of Day 32 hESC-derived photoreceptor progenitors was normal following transplantation. **(C)** Dot plot quantification analysis showing the expression of photoreceptor progenitor/ rod specific genes in H1, HADC106, H9 and HS980 embryonic cell lines respectively. In each dot plot for each marker gene, colour refers to its average gene expression calculated across all cells within a specific cluster and the size of the dot indicates the percentage of cells expressing the marker gene.

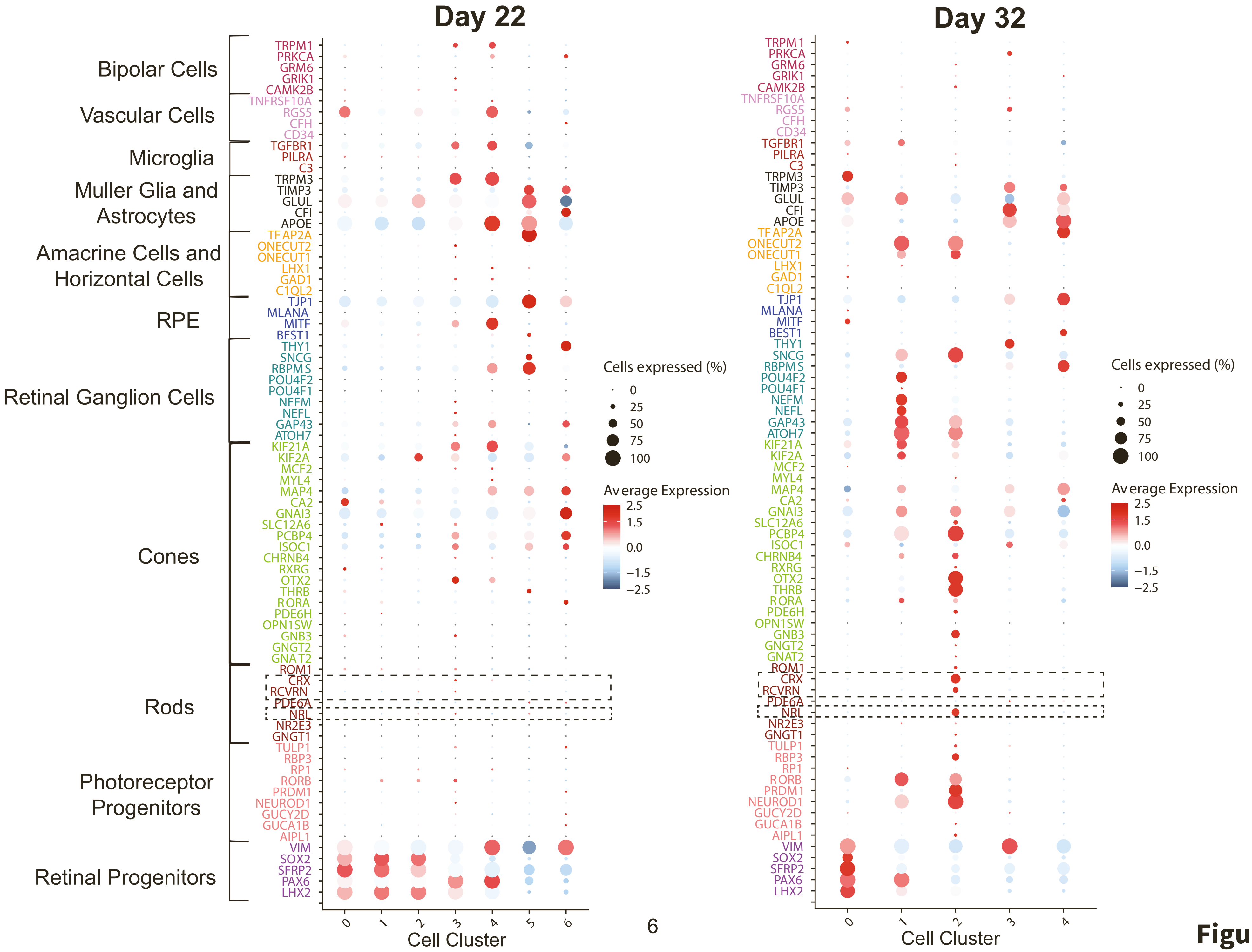

Day 22
Day 32
Bipolar Cells
Vascular Cells
Microglia
Muller Glia and Astrocytes
Amacrine Cells and Horizontal Cells
RPE
Retinal Ganglion Cells
Cones
Rods
Photoreceptor Progenitors
Retinal Progenitors
TRPM1
PRKCA
GRM6
GRIK1
CAMK2B
TNFRSF10A
RGS5
CFH
CD34
TGFBR1
PILRA
C3
TRPM3
TIMP3
GLUL
CFI
APOE
TFAP2A
ONECUT2
ONECUT1
LHX1
GAD1
C1QL2
TJP1
MLANA
MITF
BEST1
THY1
SNCG
RBPMS
POU4F2
POU4F1
NEFM
NEFL
GAP43
ATOH7
KIF21A
KIF2A
MCF2
MYL4
MAP4
CA2
GNAI3
SLC12A6
PCBP4
ISOC1
CHRNB4
RXRG
OTX2
THRB
RORA
PDE6H
OPN1SW
GNB3
GNGT2
GNAT2
ROM1
CRX
RCVRN
PDE6A
NRL
NR2E3
GNGT1
TULP1
RBP3
RP1
RORB
PRDM1
NEUROD1
GUCY2D
GUCA1B
AIPL1
VIM
SOX2
SFRP2
PAX6
LHX2
Cells expressed (%)
0
25
50
75
100
Average Expression
2.5
1.5
0.0
−1.5
−2.5
Cell Cluster

6

Figure S3

Figure S3. Dot plot quantification analysis showing that higher percentage Day 32 hESC-derived photoreceptor progenitors co-express the photoreceptor markers *Nrl*, *CRX* and *RCVRN* (as boxed) as compared to Day 22 cells. For each marker gene, colour refers to its average gene expression calculated across all cells within a specific cluster and the size of the dot indicates the percentage of cells expressing the marker gene.

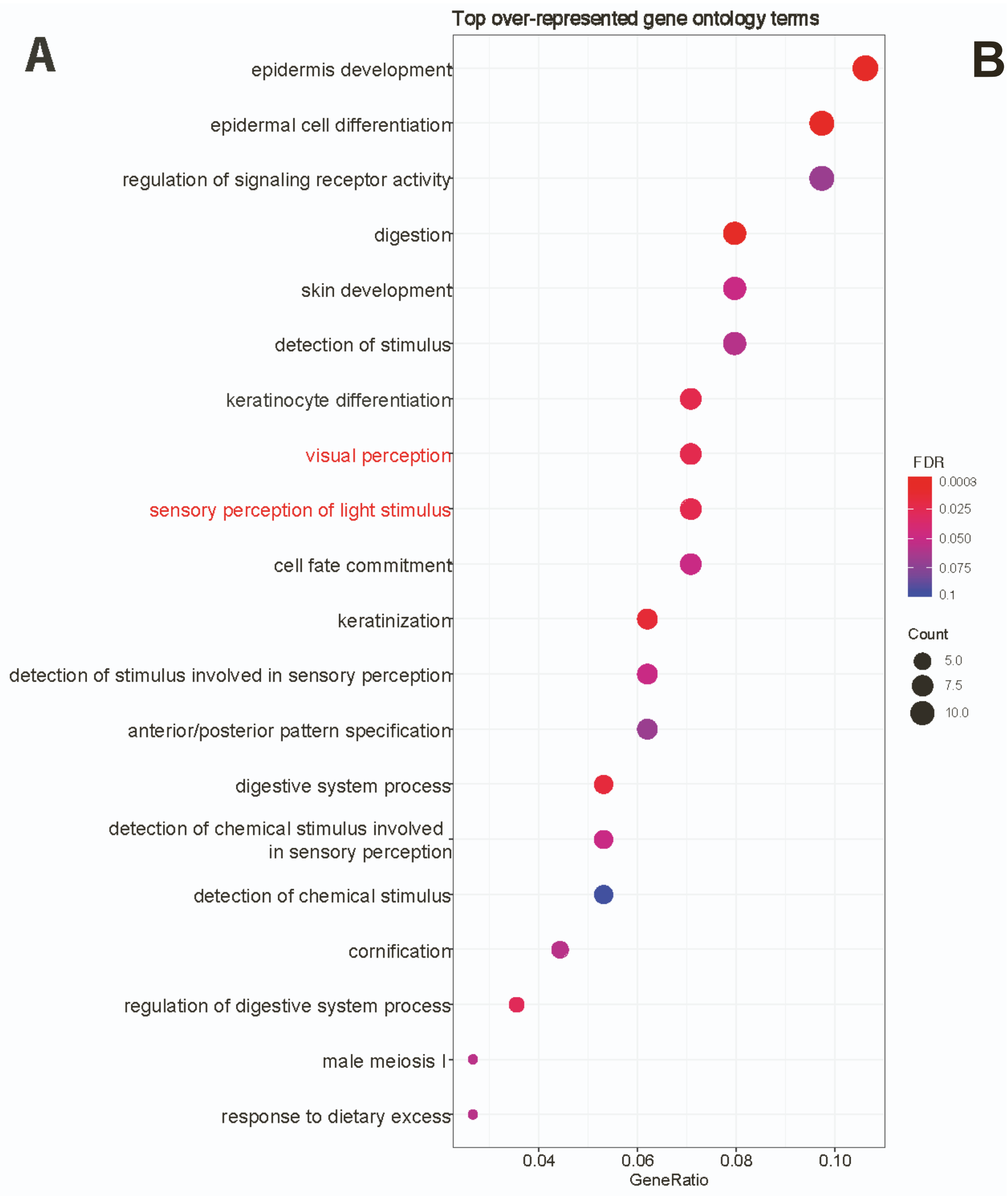
A
Top over-represented gene ontology terms
epidermis development
epidermal cell differentiation
regulation of signaling receptor activity
digestion
skin development
detection of stimulus
keratinocyte differentiation
visual perception
sensory perception of light stimulus
cell fate commitment
keratinization
detection of stimulus involved in sensory perception
anterior/posterior pattern specification
digestive system process
detection of chemical stimulus involved in sensory perception
detection of chemical stimulus
cornification
regulation of digestive system process
male meiosis I
response to dietary excess
0.04
0.06
0.08
0.10
GeneRatio
FDR
0.0003
0.025
0.050
0.075
0.1
Count
5.0
7.5
10.0

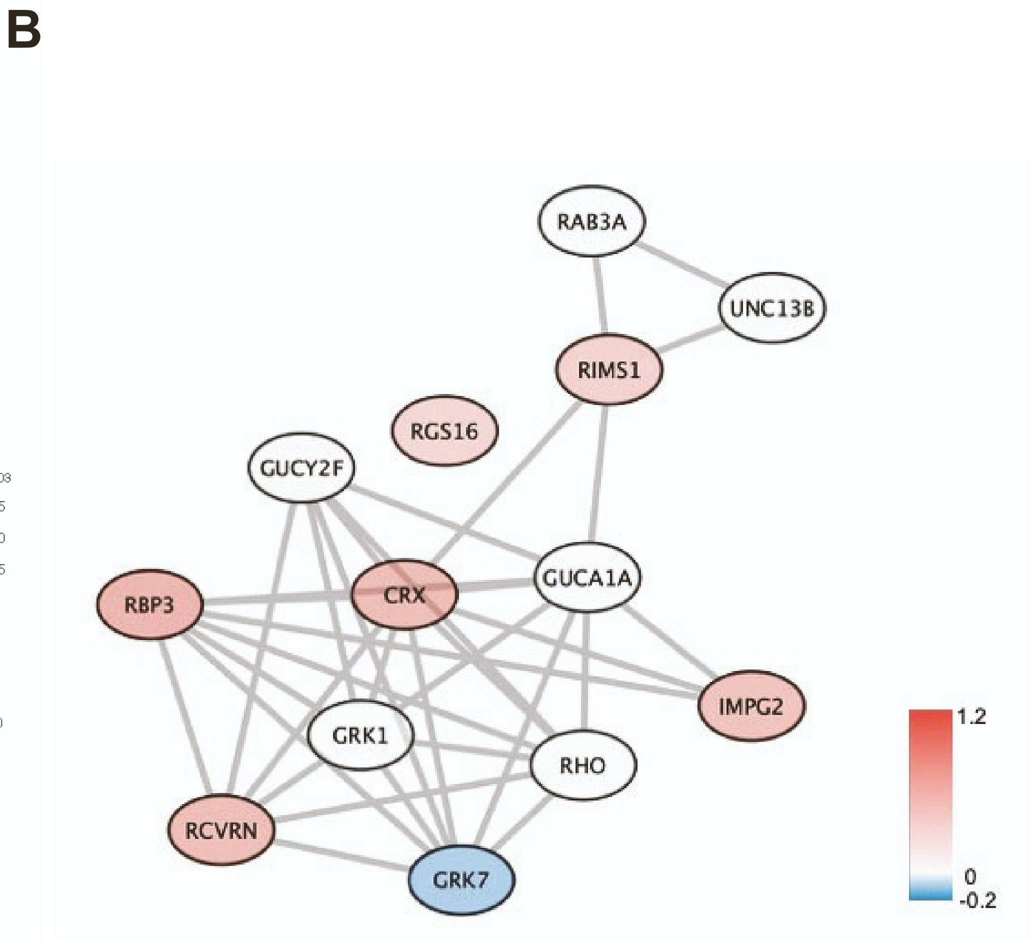
B
RAB3A
UNC13B
RIMS1
RGS16
GUCY2F
GUCA1A
RBP3
CRX
IMPG2
GRK1
RHO
RCVRN
GRK7
1.2
0
-0.2

Figure S4

Figure S4. Laminin α5 chain-mediated gene expression. (A) Over-representation analysis of Gene Ontology (GO) terms for the differentially-expressed (DE) genes (false discovery date (FDR) < 0.05, n=140) between cells cultured on LN523 and LN323 matrices at Day 32. The top GO biological processes overrepresented in the DE genes (FDR<0.1) are shown. Counts are the number of DE genes presented in the network, and GeneRatio is the percentage of the counts against the total number of genes in the network. The terms "visual perception" and "sensory perception of light stimulus" are highlighted in red text (FDR = 0.022). **(B)** Protein-protein interaction (PPI) network constructed based on the DE genes (LN523 vs LN323 at Day 32, FDR<0.05) in visual perception pathway using STRING db version 11.0. The genes are colour-coded based on the log2 fold change of DE test.

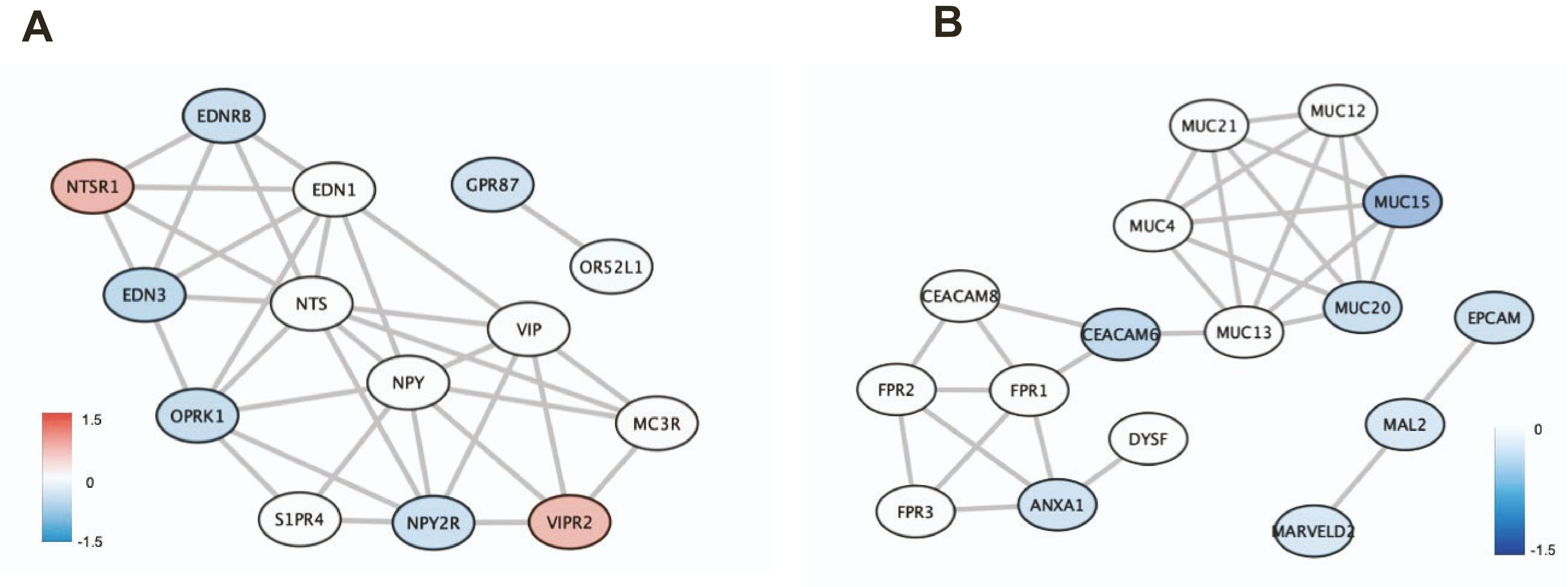
A
B
EDNRB
NTSR1
EDN1
GPR87
OR52L1
EDN3
NTS
VIP
NPY
OPRK1
MC3R
S1PR4
NPY2R
VIPR2
1.5
0
-1.5
MUC21
MUC12
MUC15
MUC4
CEACAM8
CEACAM6
MUC13
MUC20
EPCAM
FPR2
FPR1
DYSF
MAL2
FPR3
ANXA1
MARVELD2
0
-1.5

Figure S5

Figure S5. Laminin γ3 chain-mediated gene expression. Over-representation analysis of Gene Ontology (GO) terms for the differentially-expressed (DE) genes (FDR < 0.05, n=241) between Day 32 cells cultured on LN523 and LN521 matrices. The genes in top GO molecular function term G protein-coupled receptor activity (FDR = 0.063) is show in **(A)** by Protein-protein interaction (PPI) network, and the genes in top GO cell component term apical plasma membrane (FDR=0.053) is shown in **(B)** using PPI network.

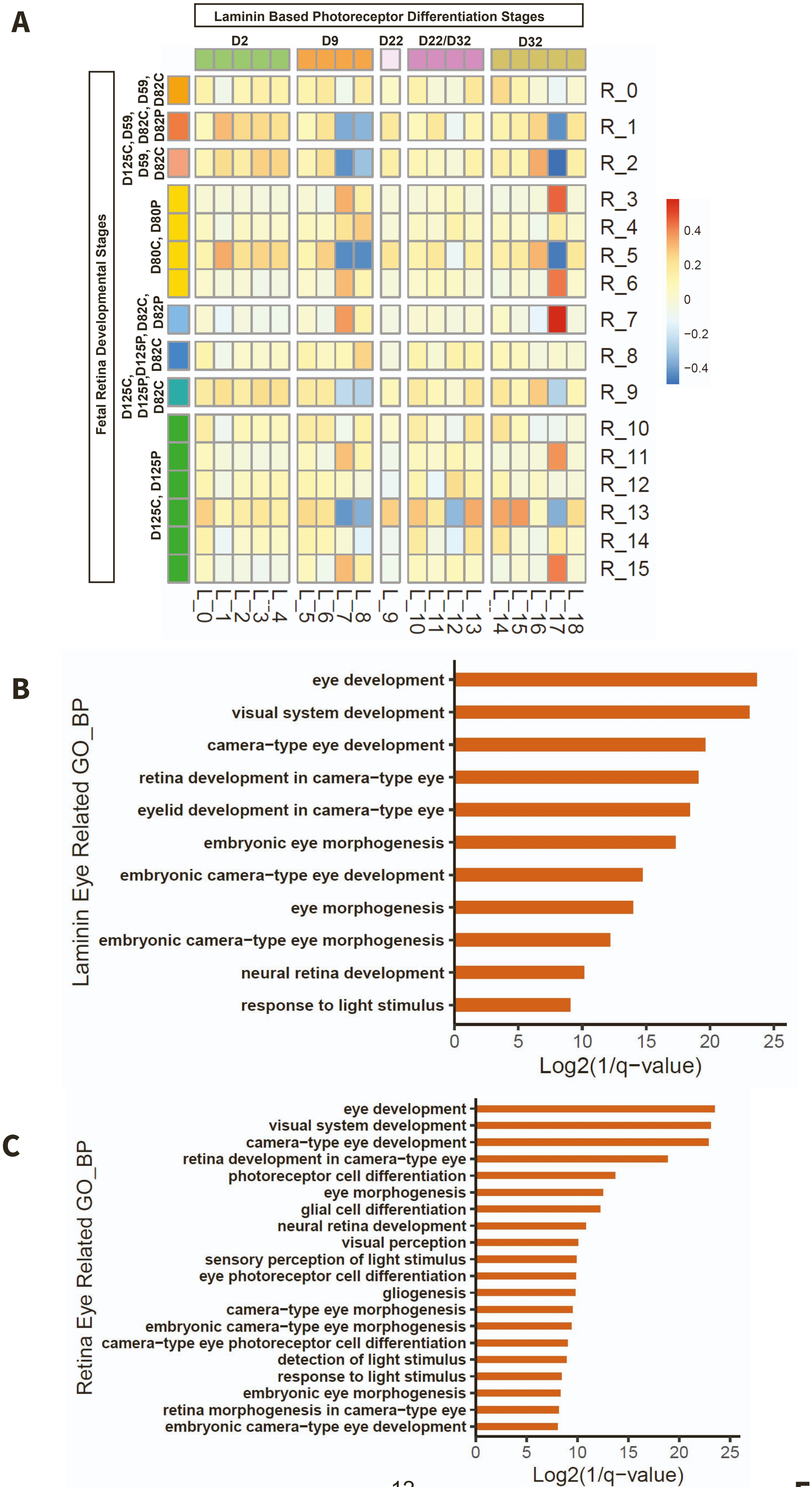
A
Laminin Based Photoreceptor Differentiation Stages
D2
D9
D22
D22/D32
D32
Fetal Retina Developmental Stages
D125C, D59, D59, D82C, D82C
D59, D82C, D82P D82C
D80C, D80P
D125C, D125P, D125P, D82C, D82C D82P
D82C
D125C, D125P
R_0
R_1
R_2
R_3
R_4
R_5
R_6
R_7
R_8
R_9
R_10
R_11
R_12
R_13
R_14
R_15
L_0
L_1
L_2
L_3
L_4
L_5
L_6
L_7
L_8
L_9
L_10
L_11
L_12
L_13
L_14
L_15
L_16
L_17
L_18
0.4
0.2
0
−0.2
−0.4
B
Laminin Eye Related GO_BP
eye development
visual system development
camera−type eye development
retina development in camera−type eye
eyelid development in camera−type eye
embryonic eye morphogenesis
embryonic camera−type eye development
eye morphogenesis
embryonic camera−type eye morphogenesis
neural retina development
response to light stimulus
0
5
10
15
20
25
Log2(1/q−value)
C
Retina Eye Related GO_BP
eye development
visual system development
camera−type eye development
retina development in camera−type eye
photoreceptor cell differentiation
eye morphogenesis
glial cell differentiation
neural retina development
visual perception
sensory perception of light stimulus
eye photoreceptor cell differentiation
gliogenesis
camera−type eye morphogenesis
embryonic camera−type eye morphogenesis
camera−type eye photoreceptor cell differentiation
detection of light stimulus
response to light stimulus
embryonic eye morphogenesis
retina morphogenesis in camera−type eye
embryonic camera−type eye development
0
5
10
15
20
25
Log2(1/q−value)

Figure S6

Figure S6. Comparisons of retinal laminin-based photoreceptor differentiation with fetal retina. **(A)** Pairwise correlations of single-cell transcriptomic clusters comparing between photoreceptor progenitors differentiated on LN523+LN521 culture matrix or during fetal retinogenesis, calculated for all genes. The color bar represents the spearman rank correlation score. Vision-related GO terms of the differentially-expressed marker genes in **(B)** photoreceptor progenitors differentiated on LN523+LN521 culture matrix and **(C)** during fetal retinogenesis.

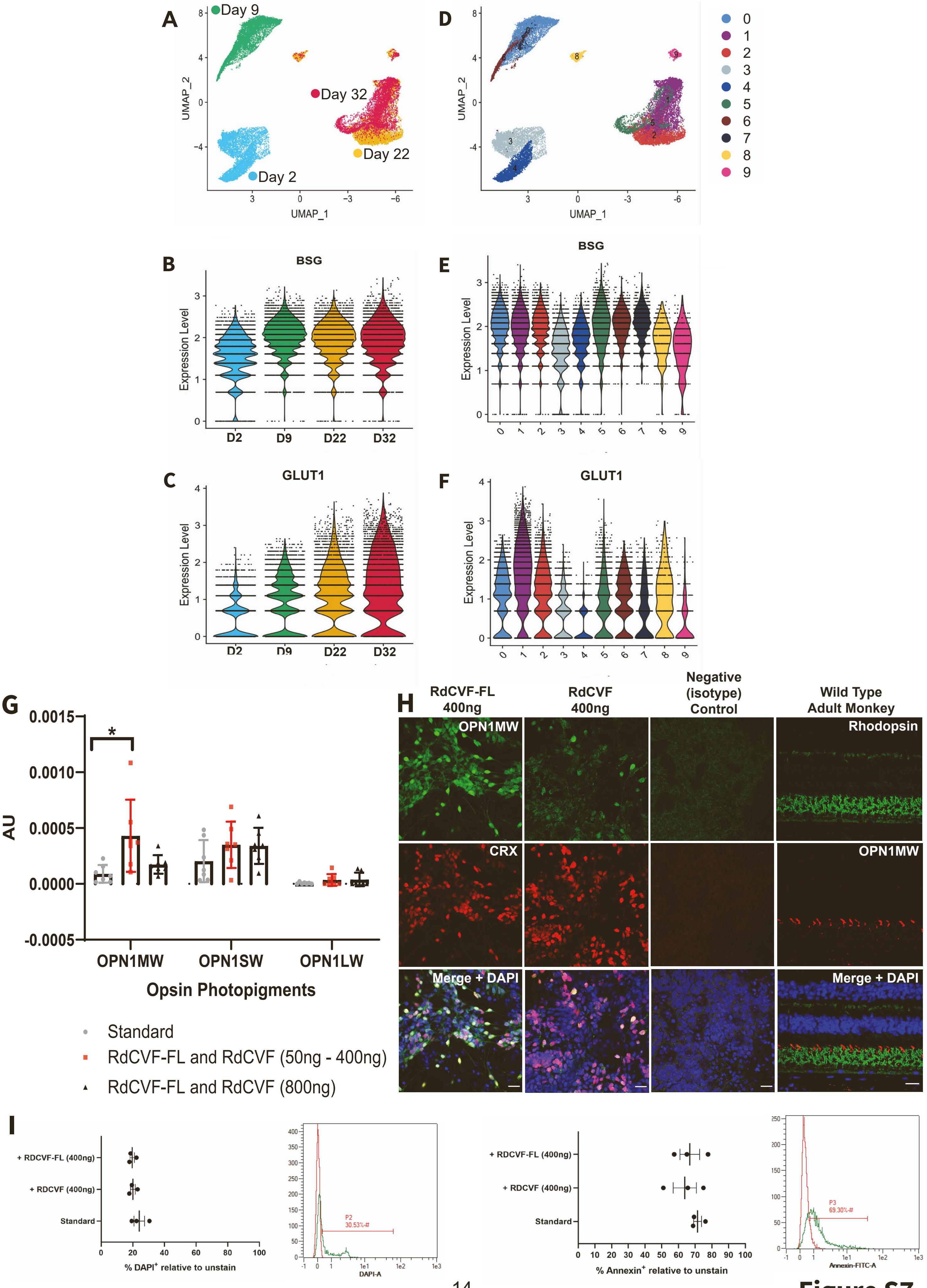
A
Day 9
Day 32
Day 22
Day 2
UMAP_1
UMAP_2
D
B
BSG
Expression Level
D2
D9
D22
D32
E
BSG
C
GLUT1
F
GLUT1
G
AU
OPN1MW
OPN1SW
OPN1LW
Opsin Photopigments
Standard
RdCVF-FL and RdCVF (50ng - 400ng)
RdCVF-FL and RdCVF (800ng)
H
RdCVF-FL 400ng
RdCVF 400ng
Negative (isotype) Control
Wild Type Adult Monkey
OPN1MW
Rhodopsin
CRX
OPN1MW
Merge + DAPI
Merge + DAPI
I
+ RDCVF-FL (400ng)
+ RDCVF (400ng)
Standard
% DAPI+ relative to unstain
P2 30.53%-#
DAPI-A
% Annexin+ relative to unstain
P3 69.30%-#
Annexin-FITC-A

Figure S7

Figure S7. RdCVF increases cone subtype expression during LN523-based photoreceptor differentiation. (A) UMAP and violin plot analyses showing increased expression of **(B)** Basagin (*BSG*) and **(C)** Glucose transporter 1 (*GLUT1*, also known as *SLC2A1*) on Day 9, Day 22, and Day 32 of hESC-derived photoreceptor progenitors as compared to Day 2. **(D)** UMAP and violin plots showing **(E)** *BSG* and **(F)** *GLUT1/ SLC2A1* are highly expressed at Day 32, with *BSG* more enriched in Day 32 *CRX* positive cell cluster. **(G)** qPCR analyses showing higher expression of *OPN1MW* as compared to *OPN1LW* expression in Day 32 hESC-derived photoreceptor progenitors cultured with RdCVF/FL ($p = 0.005$, $n = 7$). **(H)** Immunofluorescence analyses showing expression of OPN1MW in Day 32 hESC-derived photoreceptor progenitors cultured with RdCVF/FL, with monkey retina sections as positive control. Scale bars: 20 µm. **(I)** Comparison of FACS Analysis between Day 32 hESC-derived photoreceptor progenitors un-treated (control) with cells treated with RdCVF/RdCVFL Representative flow cytometry histograms depicting % of DAPI and AnnexinV stained cells.
